# Medicinal cannabis for psychiatry-related conditions: an overview of current Australian prescribing

**DOI:** 10.3389/fphar.2023.1142680

**Published:** 2023-06-06

**Authors:** Elizabeth A. Cairns, Melissa J. Benson, Miguel A. Bedoya-Pérez, Sara L. Macphail, Adith Mohan, Rhys Cohen, Perminder S. Sachdev, Iain S. McGregor

**Affiliations:** ^1^ The Lambert Initiative for Cannabinoid Therapeutics, The University of Sydney, Sydney, NSW, Australia; ^2^ Faculty of Medicine and Health, School of Pharmacy, The University of Sydney, Sydney, NSW, Australia; ^3^ Brain and Mind Centre, The University of Sydney, Camperdown, NSW, Australia; ^4^ Faculty of Science, School of Psychology, The University of Sydney, Sydney, NSW, Australia; ^5^ Centre for Healthy Brain Ageing (CHeBA), Discipline of Psychiatry and Mental Health, Faculty of Medicine and Health, University of New South Wales, Sydney, NSW, Australia; ^6^ Neuropsychiatric Institute, The Prince of Wales Hospital, Randwick, NSW, Australia

**Keywords:** medicinal cannabis, Australia, psychiatry, anxiety disorders, prescribing, medicinal cannabis use

## Abstract

**Objective:** Evidence is accumulating that components of the *Cannabis sativa* plant may have therapeutic potential in treating psychiatric disorders. Medicinal cannabis (MC) products are legally available for prescription in Australia, primarily through the Therapeutic Goods Administration (TGA) Special Access Scheme B (SAS-B). Here we investigated recent prescribing practices for psychiatric indications under SAS-B by Australian doctors.

**Methods:** The dataset, obtained from the TGA, included information on MC applications made by doctors through the SAS-B process between 1st November 2016 and 30th September 2022 inclusive. Details included the primary conditions treated, patient demographics, prescriber location, product type (e.g., oil, flower or capsule) and the general cannabinoid content of products. The conditions treated were categorized according to the Diagnostic and Statistical Manual of Mental Disorders, 5th edition, text revision (DSM-5-TR). Trends in prescribing for conditions over time were analyzed via polynomial regression, and relationships between categorical variables determined via correspondence analyses.

**Results:** Approximately 300,000 SAS-B approvals to prescribe MC had been issued in the time period under investigation. This included approvals for 38 different DSM-5-TR defined psychiatric conditions (33.9% of total approvals). The majority of approvals were for anxiety disorders (66.7% of psychiatry-related prescribing), sleep-wake disorders (18.2%), trauma- and stressor-related disorders (5.8%), and neurodevelopmental disorders (4.4%). Oil products were most prescribed (53.0%), followed by flower (31.2%) and other inhaled products (12.4%). CBD-dominant products comprised around 20% of total prescribing and were particularly prevalent in the treatment of autism spectrum disorder. The largest proportion of approvals was for patients aged 25–39 years (46.2% of approvals). Recent dramatic increases in prescribing for attention deficit hyperactivity disorder were identified.

**Conclusion:** A significant proportion of MC prescribing in Australia is for psychiatry-related indications. This prescribing often appears somewhat “experimental”, given it involves conditions (e.g., ADHD, depression) for which definitive clinical evidence of MC efficacy is lacking. The high prevalence of THC-containing products being prescribed is of possible concern given the psychiatric problems associated with this drug. Evidence-based clinical guidance around the use of MC products in psychiatry is lacking and would clearly be of benefit to prescribers.

## Introduction

Cannabis is a drug that has had a somewhat troubled relationship with psychiatry. The classic writings of Moreau de Tours described how hashish can precipitate an acute psychotic state (Abel, 2005), and numerous subsequent studies have probed the complex relationship between chronic cannabis use and schizophrenia (Arseneault et al., 2002; D'Souza et al., 2022; Hill, 2015; National Academies of Sciences, 2017; Pasman et al., 2018). Recent analyses suggest that high frequency use of more potent cannabis may be a risk factor for schizophrenia, although the debate continues (Colizzi et al., 2020; D'Souza et al., 2022; Di Forti et al., 2019). A modest non-causal association between cannabis use and depression is also widely proposed (Gorfinkel et al., 2020; Hodgson et al., 2020; Onaemo et al., 2021). Delayed initiation of cannabis use by adolescents most likely benefits their mental health, although claims of cannabis use causing irreversible adolescent brain damage have been largely debunked, though some uncertainty remains (DeLisi, 2008; Weiland et al., 2015; Fischer et al., 2022).

Sitting somewhat uncomfortably against this backdrop is the growing access to cannabis for medicinal purposes across many jurisdictions. Medicinal cannabis (MC) in Australia became legally available to prescribe in November 2016, enabling patient access to quality standardized medicinal cannabis products, even though they fall outside the “Australian Register of Therapeutic Goods”. Access is regulated by the Therapeutic Goods Administration (TGA), with the main access mechanism known as the Special Access Scheme B (SAS-B), whereby healthcare practitioners apply to the TGA to prescribe a specific type of product to an individual patient with a specific indication (reviewed in MacPhail et al., 2022).

Patient access to medical cannabis in Australia was initially very slow (MacPhail et al., 2022), due to a cumbersome application processes and high cost of products on offer (Lintzeris et al., 2022). An additional problem was that medical professionals felt relatively uneducated about medicinal cannabis products, regulatory frameworks, and therapeutic value despite ever-increasing patient interest (Karanges et al., 2018; Benson et al., 2020; Bawa et al., 2022). The past 2 years, however, has seen a dramatic rise in prescribing due to streamlined application processes, improved doctor education and a rise in cannabis-access clinics that specialize in MC prescribing (Karanges et al., 2018; Benson et al., 2020; Bawa et al., 2022). Accordingly, at the time of writing, the TGA has now issued more than 360,000 approvals for medicinal cannabis access in Australia through the SAS-B scheme. An increasing number of prescriptions are also now being made under the “Authorised Prescriber” scheme which provides a blanket approval for a healthcare practitioner to prescribe products to patients with a specific indication (Therapeutic Goods Administration, 2022a).

There are now more than 360 distinct medicinal cannabis products currently accessible to patients involving many different formulations, routes of administration, and cannabinoid profiles (Therapeutic Goods Administration, 2022b). The majority are oral formulations (oils, sprays, capsules) although there has been a recent surge in the use of plant cannabis products (also known as “flower” or “flos”) (MacPhail et al., 2022). The TGA identifies five different categories of product according to their ∆^9^-tetrahydrocannabinol (THC) and cannabidiol (CBD) content, with around one third of available products primarily containing CBD (Therapeutic Goods Administration, 2022b).

The optimal clinical use of different products and cannabinoid profiles across different conditions is still uncertain. Recent analyses lend some support to the use of THC in treating chronic pain, multiple sclerosis spasticity, anorexia/cachexia and Tourette syndrome (National Academies of Sciences, 2017; Therapeutic Goods Administration, 2019) while evidence supports CBD efficacy in the treatment of epilepsy (Devinsky et al., 2017; Devinsky et al., 2018). CBD may attenuate some of the intoxicating and other adverse psychological effects of THC, although the evidence for this is mixed (Arkell et al., 2019; Freeman et al., 2019; Englund et al., 2022; Hutten et al., 2022; Zamarripa et al., 2023).

CBD has generated some excitement in neurology and psychiatry with the proprietary oil-based CBD formulation “Epidyolex” now an FDA- and TGA-approved medicine for the treatment of specific intractable childhood epilepsies (Pauli et al., 2020). With antiepileptic drugs often successfully co-opted as psychiatric medications, it is perhaps not surprising to learn that CBD given either alone (Leweke et al., 2012) or as an adjunct to standard antipsychotic therapy (McGuire et al., 2018) shows some promise in the treatment of psychosis. Observational studies of patients, as well as open-label trials and small randomized-controlled trials (RCTs), indicate additional promise for CBD in the treatment of anxiety disorders (Masataka, 2019; Appiah-Kusi et al., 2020; Gulbransen et al., 2020; Berger, Amminger, et al., 2022). Preclinical evidence has suggested that CBD may curb addictions, with notable effects in animal models of methamphetamine and alcohol self-administration (Hay et al., 2018; Turna et al., 2019). These effects are currently being translated into clinical trials (e.g., NCT03248167, NCT03252756), with some recent findings suggesting beneficial utility in related substance abuse - cannabis-use disorder (Freeman et al., 2020).

Overall, despite some promise, the conclusions of recent systematic reviews are cautious around the use of cannabis-based medicines in psychiatric disorders citing the poor quality and patchy outcomes underpinning current evidence (Black et al., 2019; Bonaccorso et al., 2019; Hoch et al., 2019; Bahji et al., 2020; Botsford et al., 2020; Khan et al., 2020; Sarris et al., 2020; Kloiber et al., 2021; Stanciu et al., 2021). Establishing the therapeutic potential of cannabis-based medicines in psychiatry, therefore, remains a work in progress.

With this in mind, the current study involved analysis of the recent patterns of prescribing medicinal cannabis within Australia as it pertains to psychiatry-related conditions. Through information available through the TGA on SAS-B approvals, we examined the extent to which these products are being accessed via current schemes for psychiatry-related conditions, and relevant patient demographics and product characteristics.

## Materials and methods

### TGA approvals dataset

Anonymous de-identified data were obtained from the TGA through a Freedom of Information (FOI) request, informed by previous datasets (FOI 2013, 2250, 2274, 2370, 2419, 3653). Data were released from the TGA on 18th November 2022, and provided information around all SAS-B applications submitted by clinicians between 1st January 2016 and 30th September 2022 (*n* = 297,409). Applications “awaiting decision”, “cancelled/withdrawn”, or “rejected” were not included in the analyses (*n* = 4,662) or those with applications dated prior to November 2016 (*n* = 13).

### Data preparation

Data were received in a Microsoft Excel file. As in our previous analysis of SAS-B prescribing trends (MacPhail et al., 2022), the indication noted by clinicians in their SAS-B applications were not systematic and so required recoding. Indications were first coded according to the International Statistical Classification of Diseases and Related Health Problems 10th Revision (ICD-10; WHO, version 2019—English). Where required, ambiguous indications were assigned the nearest possible indication. They were then further categorized according to the Diagnostic and Statistical Manual of Mental Disorders, 5th edition, Text Revision (DSM-5-TR), and verified by two independent practicing psychiatrists (Supplementary Table S1).

Products were grouped into 11 types (capsules, extracts, crystal, flower, inhaled, lozenge, oil, spray, tablet, topical, wafer; Supplementary Table S2). The dataset contained little information that would allow an accurate analysis of the dose and/or specific medicinal cannabis product being used other than whether the product fell within Schedule 4 (≥98% CBD of total cannabinoid content) or Schedule 8 (containing ≥2% THC of total cannabinoid content) as specified by the Standard for the Uniform Scheduling of Medicines and Poisons (SUSMP). Prescriber specialty was not clear in the dataset: prior to November 2021 prescribers could volunteer their specialty as part of the application process but were not obliged to do so.

Data on patient ages were collected and were grouped for the analysis according to stratifications from the Australian Bureau of Statistics, with an additional separation of ages 10–24 to distinguish those below 18 years of age. Population data were also obtained from this source (Australian Bureau of Statistics, 2022).

### Statistical analysis

Data were analyzed with general descriptive analyses and, where described, best fit using non-linear regression models and correspondence analyses, as previously reported (MacPhail et al., 2022). Prior to non-linear regression analyses, data were processed using “tidyverse” (Wickham et al., 2019), “padr” (Thoen, 2020) and “dplyr” (Wickham et al., 2021) packages. Non-linear regressions were performed using “MASS” (Venables & Ripley, 2002), plotted with “ggplot” (Wickham, 2016), “cowplot” (Wilke, 2020) and “ggpubr” (Kassambara, 2020). Appropriate error distribution for each regression fit (i.e., Poisson or Negative binomial) was determined via Residuals plots and Pearson’s dispersion test. Fits of 1st, 2nd, 3rd, and 4th-degree polynomials were assessed via stepwise comparison of the Corrected Akaike Information Criterion (AICc) using “MuMIn” (Bartoń, 2020). Δm was calculated between models, excluding models with Δm >2 as having substantially less support (Burnham & Anderson, 2002). To estimate the goodness of fit, R^2^ was calculated for each of the best-fitted regressions by the equation: 1—deviance/residual deviance, and classified according to Moore and Kirkland (2013). Averages are listed as means ± standard error unless otherwise specified.

Associations between variables were investigated by constructing a contingency table and performing a correspondence analysis using the “Factoshiny” package (Vaissie et al., 2021). Statistics that deviated from the expected values of independence were reported in the text. Chi-squared tests were used on nominal variables through the package “stats” (R Core Team, 2022), and asymptotic linear-by-linear association tests were used on ordinal variables (i.e., age groups) through the package “coin” (Hothorn et al., 2006). This analysis is used to provide insight into overall differentiation of variables (distance from origin), similarity between variables of the same type (e.g., between two different product types; proximity), and association between variables of different types (e.g., between an indication and product; angle between the vectors connecting variables to the origin).

## Results

### Overall trends

The TGA approved 297,409 SAS-B applications for medicinal cannabis between November 2019 and September 2022 (Table 1). Psychiatric indications represented 33.9% (*n* = 100,666) of total approvals and included two out of the top three indications in the dataset [pain, *n* = 164,055 (55.2% of total prescribing); anxiety, *n* = 67,095 (22.6%); and sleep disorders, *n* = 11,202 (3.8%)].

**TABLE 1:** Overview of SAS-B approvals for psychiatric and non-DSM indications by sex and age.

	Sex^a^			Age						
	Total (%^b^)	Female (%^c^)	Male (%^c^)	0–9 (%^d^)	10–17 (%^d^)	18–24 (%^d^)	25–39 (%^d^)	40–54 (%^d^)	55–74 (%^d^)	>74 (%^d^)
Non-DSM	196,743 (66.2)	88,828 (45.1)	107,336 (54.6)	733 (0.4)	924 (0.5)	6,501 (3.3)	49,200 (25.0)	57,942 (29.5)	58,537 (29.8)	22,896 (11.6)
Anxiety Disorders	67,133 (22.6)	23,911 (35.6)	42,964 (64)	165 (0.2)	741 (1.1)	8,123 (12.1)	33,116 (49.3)	17,063 (25.4)	6,782 (10.1)	1,141 (1.7)
Anxiety	67,095 (22.6)	23,894 (35.6)	42,943 (64.0)	165 (0.2)	741 (1.1)	8,117 (12.1)	33,101 (49.3)	17,051 (25.4)	6,778 (10.1)	1,140 (1.7)
GAD	19 (0.0)	10 (52.6)	9 (47.4)	0 (0)	0 (0)	3 (15.8)	6 (31.6)	6 (31.6)	4 (21.1)	0 (0)
Panic disorder	10 (0.0)	5 (50.0)	5 (50.0)	0 (0)	0 (0)	2 (20.0)	4 (40.0)	3 (30.0)	0 (0)	1 (10.0)
SAD	9 (0.0)	2 (22.2)	7 (77.8)	0 (0)	0 (0)	1 (11.1)	5 (55.6)	3 (33.3)	0 (0)	0 (0)
Sleep-Wake Disorders	18,321 (6.2)	5,905 (32.2)	12,379 (67.6)	27 (0.1)	86 (0.5)	1,528 (8.3)	7,429 (40.5)	5,481 (29.9)	3,275 (17.9)	491 (2.7)
Sleep disorder	11,202 (3.8)	3,495 (31.2)	7,698 (68.7)	14 (0.1)	47 (0.4)	1,031 (9.2)	4,814 (43.0)	3,263 (29.1)	1,790 (16.0)	240 (2.1)
Insomnia	6,877 (2.3)	2,308 (33.6)	4,544 (66.1)	13 (0.2)	37 (0.5)	491 (7.1)	2,591 (37.7)	2,166 (31.5)	1,365 (19.8)	213 (3.1)
RLS	239 (0.1)	101 (42.3)	135 (56.5)	0 (0)	2 (0.8)	6 (2.5)	23 (9.6)	51 (21.3)	119 (49.8)	38 (15.9)
Narcolepsy	1 (0.0)	1 (100)	0 (0)	0 (0)	0 (0)	0 (0)	0 (0)	0 (0)	1 (100)	0 (0)
Hypersomnia	1 (0.0)	0 (0)	1 (100)	0 (0)	0 (0)	0 (0)	1 (100)	0 (0)	0 (0)	0 (0)
Parasomnia	1 (0.0)	0 (0)	1 (100)	0 (0)	0 (0)	0 (0)	0 (0)	1 (100)	0 (0)	0 (0)
Trauma- and Stressor-Related Disorders										
PTSD	5,799 (1.9)	2,216 (38.2)	3,546 (61.1)	0 (0)	18 (0.3)	419 (7.2)	2,372 (40.9)	2,141 (36.9)	799 (13.8)	50 (0.9)
Neurodevelopmental Disorders	4,450 (1.5)	961 (21.6)	3,455 (77.6)	550 (12.4)	1,138 (25.6)	807 (18.1)	1,550 (34.8)	330 (7.4)	67 (1.5)	7 (0.2)
ASD	2,206 (0.7)	522 (23.7)	1,667 (75.6)	480 (21.8)	931 (42.2)	389 (17.6)	330 (15.0)	62 (2.8)	13 (0.6)	1 (0.0)
ADHD	2,078 (0.7)	392 (18.9)	1,672 (80.5)	66 (3.2)	169 (8.1)	384 (18.5)	1,170 (56.3)	247 (11.9)	38 (1.8)	3 (0.1)
Tourette’s syndrome	163 (0.1)	47 (28.8)	113 (69.3)	1 (0.6)	38 (23.3)	34 (20.9)	50 (30.7)	21 (12.9)	16 (9.8)	3 (1.8)
Intellectual impairment	3 (0.0)	0 (0)	3 (100)	3 (100)	0 (0)	0 (0)	0 (0)	0 (0)	0 (0)	0 (0)
Depressive Disorders	4,003 (1.3)	1,353 (33.8)	2,639 (65.9)	4 (0.1)	20 (0.5)	483 (12.1)	1,785 (44.6)	1,121 (28)	529 (13.2)	59 (1.5)
Depression	3,247 (1.1)	1,103 (34)	2,134 (65.7)	0 (0)	17 (0.5)	390 (12)	1,474 (45.4)	884 (27.2)	429 (13.2)	51 (1.6)
Mood disorder	736 (0.2)	243 (33)	492 (66.8)	4 (0.5)	3 (0.4)	90 (12.2)	307 (41.7)	230 (31.3)	94 (12.8)	8 (1.1)
Major depression	15 (0.0)	2 (13.3)	13 (86.7)	0 (0)	0 (0)	1 (6.7)	2 (13.3)	6 (40.0)	6 (40.0)	0 (0)
PDD	5 (0.0)	5 (100)	0 (0)	0 (0)	0 (0)	2 (40.0)	2 (40.0)	1 (20.0)	0 (0)	0 (0)
Neurocognitive Disorders	428 (0.1)	252 (58.9)	176 (41.1)	0 (0)	1 (0.2)	4 (0.9)	10 (2.3)	26 (6.1)	112 (26.2)	275 (64.3)
Alzheimer’s disease	272 (0.1)	166 (61.0)	106 (39.0)	0 (0)	0 (0)	3 (1.1)	9 (3.3)	8 (2.9)	69 (25.4)	183 (67.3)
Unspecified dementia	132 (0.0)	75 (56.8)	57 (43.2)	0 (0)	1 (0.8)	1 (0.8)	0 (0)	4 (3.0)	36 (27.3)	90 (68.2)
Huntington chorea	22 (0)	9 (40.9)	13 (59.1)	0 (0)	0 (0)	0 (0)	1 (4.5)	14 (63.6)	6 (27.3)	1 (4.5)
Memory loss	1 (0)	1 (100)	0 (0)	0 (0)	0 (0)	0 (0)	0 (0)	0 (0)	1 (100)	0 (0)
Cognitive decline	1 (0)	1 (100)	0 (0)	0 (0)	0 (0)	0 (0)	0 (0)	0 (0)	0 (0)	1 (100)
Bipolar and Related Disorders										
Bipolar disorder	212 (0.1)	83 (39.2)	127 (59.9)	0 (0)	1 (0)	17 (8)	98 (46)	79 (37)	17 (8)	0 (0)
Disruptive, Impulse-Control, and Conduct Disorders	155 (0.1)	53 (34.2)	100 (64.5)	13 (8.0)	34 (22.0)	23 (15.0)	36 (23.0)	14 (9.0)	14 (9.0)	21 (14.0)
Behavior disorder	131 (0.0)	45 (34.4)	84 (64.1)	12 (9.0)	30 (23.0)	23 (18.0)	34 (26.0)	12 (9.0)	10 (8.0)	10 (8.0)
Aggressive behavior	13 (0.0)	5 (38.5)	8 (61.5)	1 (8.0)	4 (31.0)	0 (0)	0 (0)	0 (0)	3 (23.0)	5 (38.0)
Agitation	11 (0.0)	3 (27.3)	8 (72.7)	0 (0)	0 (0)	0 (0)	2 (18.0)	2 (18.0)	1 (9.0)	6 (55.0)
Substance-Related and Addictive Disorders	126 (0.0)	20 (15.9)	106 (84.1)	0 (0)	0 (0)	21 (17.0)	63 (50.0)	26 (21.0)	16 (13.0)	0 (0)
Cannabis use disorder	117 (0.0)	17 (14.5)	100 (85.5)	0 (0)	0 (0)	21 (18.0)	59 (50.0)	22 (19.0)	15 (13.0)	0 (0)
Unspecified addiction	6 (0.0)	3 (50.0)	3 (50.0)	0 (0)	0 (0)	0 (0)	3 (50.0)	3 (50.0)	0 (0)	0 (0)
Alcohol dependence	2 (0.0)	0 (0)	2 (100)	0 (0)	0 (0)	0 (0)	1 (50.0)	1 (50.0)	0 (0)	0 (0)
Tobacco use disorder	1 (0.0)	0 (0)	1 (100)	0 (0)	0 (0)	0 (0)	0 (0)	0 (0)	1 (100)	0 (0)
Schizophrenia Spectrum and Other Psychotic Disorders										
Schizophrenia	31 (0.0)	3 (9.7)	28 (90.3)	0 (0)	0 (0)	3 (10.0)	17 (55.0)	9 (29.0)	2 (6.0)	0 (0)
Obsessive-Compulsive and Related disorders										
OCD	4 (0.0)	1 (25.0)	3 (75.0)	0 (0)	0 (0)	1 (25.0)	1 (25.0)	1 (25.0)	1 (25.0)	0 (0)
Somatic Symptom and Related Disorders	2 (0.0)	2 (100)	0 (0)	0 (0)	0 (0)	0 (0)	1 (50.0)	1 (50.0)	0 (0)	0 (0)
Bruxism	1 (0.0)	1 (100)	0 (0)	0 (0)	0 (0)	0 (0)	1 (100)	0 (0)	0 (0)	0 (0)
Psychogenic seizures	1 (0.0)	1 (100)	0 (0)	0 (0)	0 (0)	0 (0)	0 (0)	1 (100)	0 (0)	0 (0)
Medication-Induced Movement Disorders and Other Adverse Effects of Medication	2 (0.0)	1 (50.0)	1 (50.0)	0 (0)	0 (0)	0 (0)	0 (0)	2 (100)	0 (0)	0 (0)
Extrapyramidal symptoms	1 (0.0)	0 (0)	1 (100)	0 (0)	0 (0)	0 (0)	0 (0)	1 (100)	0 (0)	0 (0)
Tardive dyskinesia	1 (0.0)	1 (100)	0 (0)	0 (0)	0 (0)	0 (0)	0 (0)	1 (100)	0 (0)	0 (0)
**Total**	**297,409 (100)**	**123,589 (42.0)**	**172,860 (58.1)**	**1,492 (0.5)**	**2,963 (1.0)**	**17,930 (6.0)**	**95,678 (32.2)**	**84,236 (28.3)**	**70,151 (23.6)**	**24,940 (8.4)**

^a^ Sex indeterminate/intersex/unspecified data not shown (*n* = 960 or 0.4%; 381 for psychiatric indications).

^b^ Percentage of prescribing over all indications (including non-DSM, indications).

^c^ Percentage of prescribing in each indication by sex.

^d^ Percentage prescribing in each indication by age group (age unknown not shown, *n* = 19). ADHD: attention deficit hyperactivity disorder; ASD: autism spectrum disorder; GAD: generalized anxiety disorder; OCD: Obsessive-compulsive disorder; PDD: premenstrual dysphoric disorder; PTSD: Post-traumatic stress disorder; RLS: restless legs syndrome; SAD: social anxiety disorder.

Approvals for psychiatric indications covered thirteen general DSM-5-TR categories: anxiety disorders (n = 67,133; 22.6% of total prescribing); sleep-wake disorders (*n* = 18,321; 6.2%); trauma and stressor-related disorders (*n* = 5,799; 1.9%); neurodevelopmental disorders (*n* = 4,450; 1.5%); depressive disorders (*n* = 4,003; 1.3%); neurocognitive disorders (*n* = 428, 0.1%); bipolar and related disorders (*n* = 212; 0.1%); disruptive, impulse-control, and conduct disorders (*n* = 155; 0.1%); substance-related and addictive disorders (*n* = 126; <0.1%); schizophrenia spectrum and other psychotic disorders (*n* = 31; <0.1%); obsessive-compulsive and related disorders (*n* = 4; <0.1%); somatic symptom and related disorders (*n* = 2; <0.1%); and medication-induced movement disorders and other adverse effects of medication (*n* = 2; <0.1%).

Within the thirteen general DSM-5-TR categories, approvals for 38 specific psychiatric indications were identified, 12 of which accrued more than 200 approvals (Table 1).

### Patient demographics

A larger proportion of approved applications for psychiatric indications were for males (65.1%) compared to females (34.5%; Table 1). A total of 381 applications (0.4%) that had no sex listed or were indeterminant or intersex. Approvals for psychiatric indications were primarily for younger patients, with approvals for patients <40 years representing 60.4% of total approvals. The largest proportion of approvals was for patients 25–39 years (46.2% of total psychiatry-related approvals). When normalized by overall population, this group also had the largest *per capita* prescribing for psychiatric indications (851 approvals per 100,000; Supplementary Table S3). By contrast, this age group represented only 25.0% of the total for non-psychiatric approvals. Prescribing for 25–39 year olds also represented the largest proportion of prescribing across different psychiatric categories, with a few exceptions (neurocognitive disorders; obsessive-compulsive and related disorders; and medication-induced movement disorders). Age was unknown for nine approvals for psychiatric indications.

### Prescriber location

Queensland had the largest proportion of prescribing for psychiatric indications (54.9%), which was disproportionate to the population in this state (1,071 approvals per 100,000; Supplementary Table S4)*.* Prescribing in the Northern Territory and South Australia were the lowest *per capita* (27 approvals per 100,000).

### Products prescribed and cannabinoid content

The type of products being prescribed varied across psychiatric indications (Supplementary Table S5). The top three product types were oil (*n* = 53,347; 53.0%), flower (*n* = 31,518; 31.3%), and unspecified inhaled products (“inhaled”; *n* = 12,532; 12.4%). This varied greatly by indication: for example, 93.2% of prescribing for neurocognitive disorders was for oil products, while 44.8% of prescribing for depressive disorders was for flower products.

A greater proportion of prescribed products were S8 (>2% THC content; 79.7% of total) than S4 (>98% CBD content; 20.3% of total; Table 2). This proportion varied by psychiatric indication; for example, S4 products were more commonly used for disruptive, impulse-control, and conduct disorders (38.1%), neurodevelopmental disorders (36.4%), and neurocognitive disorders (27.8%). On the other hand, S4 approvals for depressive disorders were only 12.7% of the total, while sleep-wake-disorders had only 14.4% of approvals as S4.

**TABLE 2:** SAS-B approvals by product schedule and indication. The number of approvals by SUSMP Schedule (S4 or S8) showing percentage split between S4 and S8 for each indication or indication group in brackets.

	S4 (%)	S8 (%)
Non-DSM	44,441 (22.6)	152,302 (77.4)
Anxiety Disorders	14,508 (21.6)	52,625 (78.4)
Anxiety	14,503 (21.6)	52,592 (78.4)
GAD	0 (0)	19 (100)
Panic Disorder	5 (50.0)	5 (50.0)
SAD	0 (0)	9 (100)
Sleep-Wake Disorders	2,630 (14.4)	15,691 (85.6)
Sleep disorder	1,610 (14.4)	9,592 (85.6)
Insomnia	923 (13.4)	5,954 (86.6)
RLS	95 (39.7)	144 (60.3)
Narcolepsy	0 (0)	1 (100)
Hypersomnia	1 (100)	0 (0)
Parasomnia	1 (100)	0 (0)
Trauma- and Stressor-Related Disorders		
PTSD	874 (15.1)	4,925 (84.9)
Neurodevelopmental Disorders	1,620 (36.4)	2,830 (63.6)
ASD	1,243 (56.3)	963 (43.7)
ADHD	330 (15.9)	1,748 (84.1)
Tourette’s syndrome	44 (27.0)	119 (73.0)
Intellectual impairment	3 (100)	0 (0)
Depressive Disorders	509 (12.7)	3,494 (87.3)
Depression	447 (13.8)	2,800 (86.2)
Mood disorder	58 (7.9)	678 (92.1)
Major depression	0 (0)	15 (100)
PDD	4 (80.0)	1 (20)
Neurocognitive Disorders	119 (27.8)	309 (72.2)
Alzheimer’s disease	83 (30.5)	189 (69.5)
Unspecified dementia	36 (27.3)	96 (72.7)
Huntington chorea	0 (0)	22 (100)
Memory loss	0 (0)	1 (100)
Cognitive decline	0 (0)	1 (100)
Bipolar and Related Disorders		
Bipolar disorder	35 (16.5)	177 (83.5)
Disruptive, Impulse-Control, and Conduct Disorders	59 (38.1)	96 (61.9)
Behavior disorder	56 (42.7)	75 (57.3)
Aggressive behavior	0 (0)	13 (100)
Agitation	3 (27.3)	8 (72.7)
Substance-Related and Addictive Disorders	8 (6.3)	118 (93.7)
Cannabis use disorder	7 (6.0)	110 (94.0)
Unspecified addiction	1 (16.7)	5 (83.3)
Alcohol dependence	0 (0)	2 (100)
Tobacco use disorder	0 (0)	1 (100)
Schizophrenia Spectrum and Other Psychotic Disorders		
Schizophrenia	22 (71.0)	9 (29.0)
Obsessive-Compulsive and Related disorders		
Obsessive-compulsive disorder	1 (25.0)	3 (75.0)
Somatic Symptom and Related Disorders	1 (50.0)	1 (50.0)
Bruxism	1 (100)	0 (0)
Psychogenic seizures	0 (0)	1 (100)
Medication-Induced Movement Disorders and Other Adverse Effects of Medication	1 (50.0)	1 (50.0)
Extrapyramidal symptoms	0 (0)	1 (100)
Tardive dyskinesia	1 (100)	0 (0)
**Total**	**64,828 (21.8)**	**232,581 (78.2)**

### Associations between patient profiles, indication, and products

Associations between indication, product type, and age group were investigated for psychiatric indications with >200 approvals. The contribution to variance (CoV) on each dimension and expected variances for all analyses are included in Supplementary Table S6.

**FIGURE 1:**
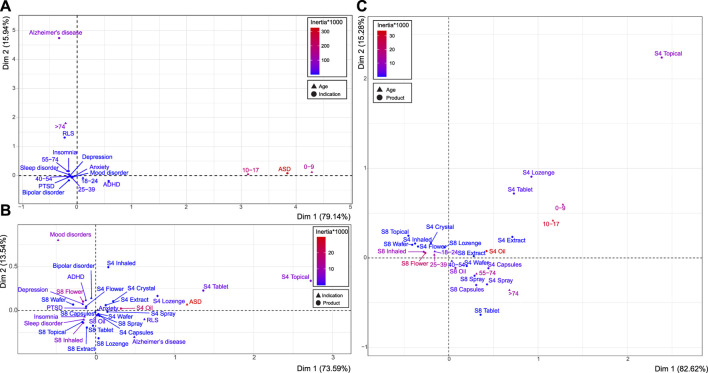
Associations between age, product schedule and type, and indication. Correspondence analyses between age and indication **(A)**, indication and product schedule and type **(B)**, and age and product schedule and type **(C)**. Deviation from independence described by the dimensions on each axis (Dim 1 and Dim 2), with the scaled contribution to the overall variance depicted by the inertia*1,000 (red to blue color gradient). See Supplementary Table S6 for related statistics.

There was a clear association between age group and indication (Z = −26.815, *p* < 0.001) (Figure 1A). Distinct conditions were ASD (CoV Dim 1 = 94.76%, inertia*1,000 = 325.731) and AD (CoV Dim 2 = 88.37%; inertia*1,000 = 62.71). AD corresponded to patients aged >74 (CoV Dim 2 = 90.56%; inertia*1,000 = 64.582), and ASD was associated with ages 0–9 (CoV Dim 1 = 39.72%; inertia*1,000 = 136.794) and 10–17 (CoV Dim 1 = 55.79%; inertia*1,000 = 191.633).

Similarly, there was an association between the condition treated and selected product format and schedule (e.g., S8 flower; χ^2^_119_ = 3,719.300, *p* < 0.001) (Figure 1B). Patients with ASD displayed the most distinct product preference (CoV Dim 1 = 75.583%, inertia*1,000 = 33.406), and was associated with S4 oil (CoV Dim 1 = 47.339%; inertia*1,000 = 19.026), S4 tablet (CoV Dim 1 = 7.495%; inertia*1,000 = 3.317), and S4 topical preparations (CoV Dim 1 = 13.826%; inertia*1,000 = 6.766). S8 flower products also represented a distinct product choice (CoV Dim 2 = 45.518%; inertia*1,000 = 11.389), which was associated with mood disorders (CoV Dim 2: 65.62%, inertia*1,000 = 6.575), amongst others. S8 inhaled formulations (CoV Dim 2 = 33.781%; inertia*1,000 = 6.493) were most associated with approvals for sleep disorders (CoV Dim 2 = 58.867%; inertia*1,000 = 8.561) and insomnia (CoV Dim 2 = 19.019%; inertia*1,000 = 4.609). A large proportion of approvals were for patients with anxiety, which represented the average profile across all product choices, as indicated by the proximity to the origin (coordinate for Dim 1 = 0.032 and Dim 2 = −0.006), as well as S8 capsules (Dim 1 = −0.006, Dim 2 = −0.020).

Product preference was also investigated in relation to patients’ age group, in which there was a clear association (Z = −10.889, *p* < 0.001) (Figure 1C). Almost all age groups had distinct product preferences (see Supplementary Table S6C). S4 oil and S8 flower represented the most distinct subgroups (CoV Dim 1 = 43.184%, inertia*1,000 = 33.748; and CoV Dim 1 = 28.237%, inertia*1,000 = 22.387, respectively). S4 oil was most associated with ages 0–9 (CoV Dim 2 = 19.133%, inertia*1,000 = 15.634) and 10–17 (CoV Dim 1 = 36.494%, inertia*1,000 = 31.445), as was S4 topical (CoV Dim 2 = 26.038%, inertia*1,000 = 7.769). S8 flower was associated with patient ages 25–39 (CoV Dim 1 = 15.922%, inertia*1,000 = 12.69), as was S8 inhaled (CoV Dim 1 = 12.861%, inertia*1,000 = 10.496). The product choice of S8 oil (CoV Dim 2 = 30.974%, inertia*1,000 = 6.315) was more commonly selected for older age groups, particularly patients aged 55–74 (CoV Dim 2 = 26.312%, inertia*1,000 = 14.93) and >74 (CoV Dim 2 = 18.912%, inertia*1,000 = 12.169).

### Trends over time

**FIGURE 2:**
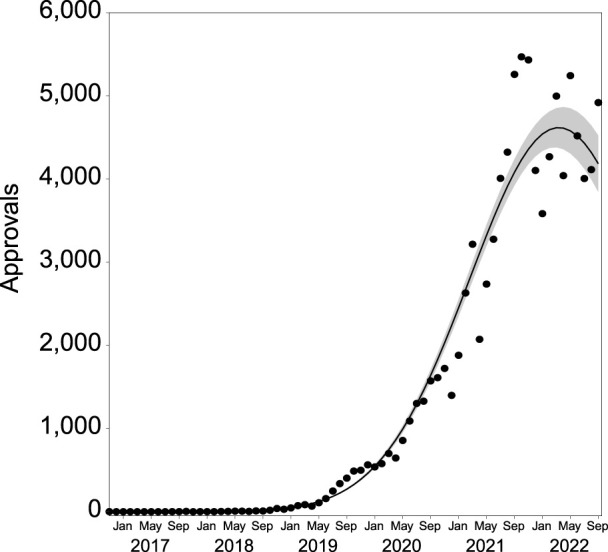
Number of SAS-B approvals per month for psychiatric indications from November 2016 to September 2022 (*n* = 100,666). The solid line represents the best fit, with shading depicting standard error of the mean (SEM).

Prescribing for psychiatric conditions has grown rapidly from November 2019, as is the case for all SAS-B prescribing (MacPhail et al., 2022). However, growth seems to be slowing or decreasing as of approximately November 2021 (2nd degree polynomial, R^2^ = 0.988, ∆m = 17,133.400; Figure 2).

**FIGURE 3:**
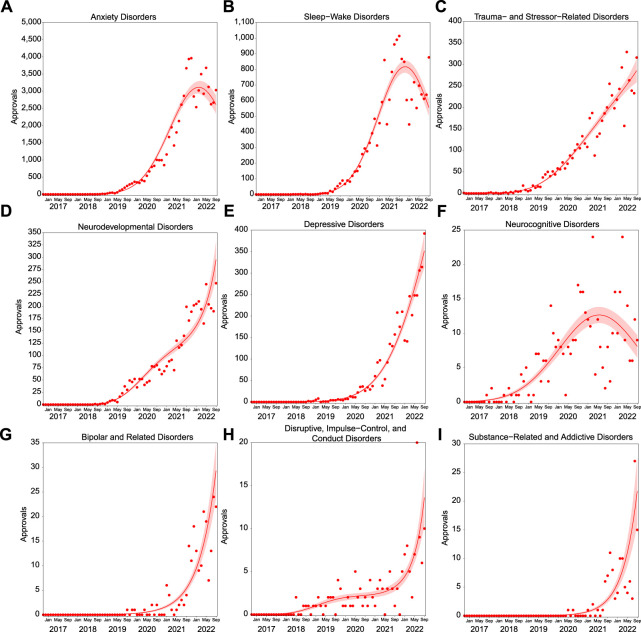
Approvals per month in psychiatric indication categories with >100 approvals followed different patterns of prescribing growth. Approvals over time for anxiety disorders [**(A)**, *n* = 67,133]; sleep wake disorders [**(B)**, *n* = 18,321], trauma- and stressor-related disorders [**(C)**, *n* = 5,799] neurodevelopmental disorders [**(D)**, *n* = 4,450]; depressive disorders [**(E)**, *n* = 4,003]; neurocognitive disorders [**(F)**, *n* = 428]; bipolar and related disorders [**(G)**, *n* = 212]; disruptive, impulse-control, and conduct disorders [**(H)**, *n* = 155]; and substance-related and addictive disorders [**(I)**, *n* = 126]. Solid lines represent the best fit, with shading depicting standard error of the mean (SEM).

**FIGURE 4:**
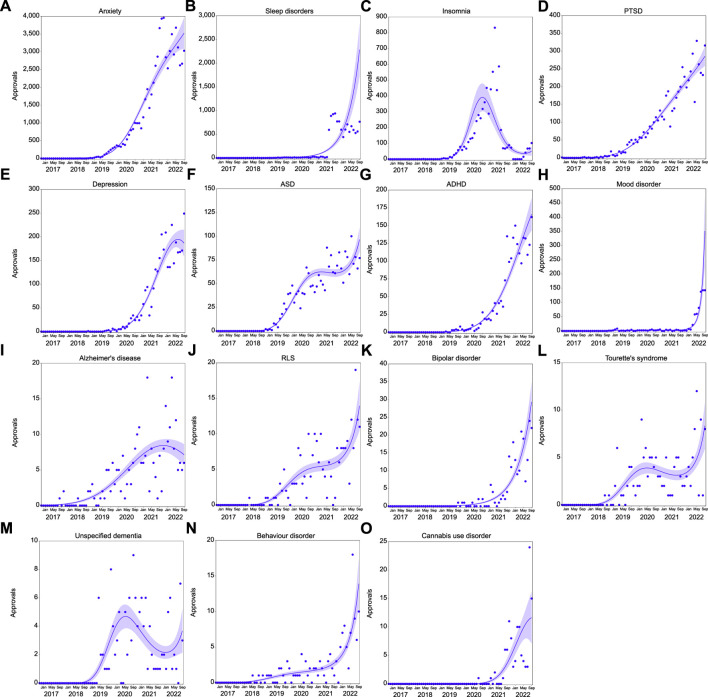
Approvals per month in psychiatric indications with >100 approvals. Approvals over time for anxiety [**(A)**, *n* = 67,095]; sleep disorder [**(B)**, *n* = 11,202)], insomnia [**(C)**, *n* = 6,877); post-traumatic stress disorder [PTSD; **(D)**, *n* = 5,799]; depression [**(E)**, *n* = 3,247]; autism spectrum disorder [ASD; **(F)**, *n* = 2,206]; attention deficit hyperactivity disorder [ADHD; **(G)**, *n* = 2,078]; mood disorder [**(H)**, *n* = 736]; Alzheimer’s disease [**(I)**, *n* = 272]; restless leg syndrome [RLS; **(J)**, *n* = 239]; bipolar disorder [**(K)**, *n* = 212]; Tourette’s syndrome [**(L)**, *n* = 163]; unspecified dementia [**(M)**, *n* = 132]; behavior disorder [**(N)**, *n* = 131]; cannabis use disorder [**(O)**, *n* = 117]. Solid lines represent the best fit, with shading depicting standard error of the mean (SEM).

Trends over time are not uniform across all psychiatric indications. Prescribing for neurodevelopmental disorders (3^rd^ degree polynomial, R^2^ = 0.975, ∆m = 30.228); depressive disorders (2nd degree polynomial, R^2^ = 0.973, ∆m = 19.527); bipolar and related disorders (1st degree polynomial, R^2^ = 0.873); disruptive and related disorders (3rd degree polynomial, R^2^ = 0.728, ∆m = 9.483); and substance-and related addictive disorders (1st degree polynomial, R^2^ = 0.862) have shown dramatic increases in approvals (Figure 3). At the individual indication level (>100 approvals), prescribing also continues to increase for sleep disorders (2nd degree polynomial, R^2^ = 0.726, ∆m = 5.805), PTSD (3rd degree polynomial, R^2^ = 0.972, ∆m = 4.642), ADHD (2nd degree polynomial, R^2^ = 0.966, ∆m = 19.397), mood disorder (3rd degree polynomial, R^2^ = 0.893, ∆m = 39.410), bipolar disorder (1st degree polynomial, R^2^ = 0.873), and behavior disorder (3rd degree polynomial, R^2^ = 0.730, ∆m = 6.371; Figure 4).

**FIGURE 5:**
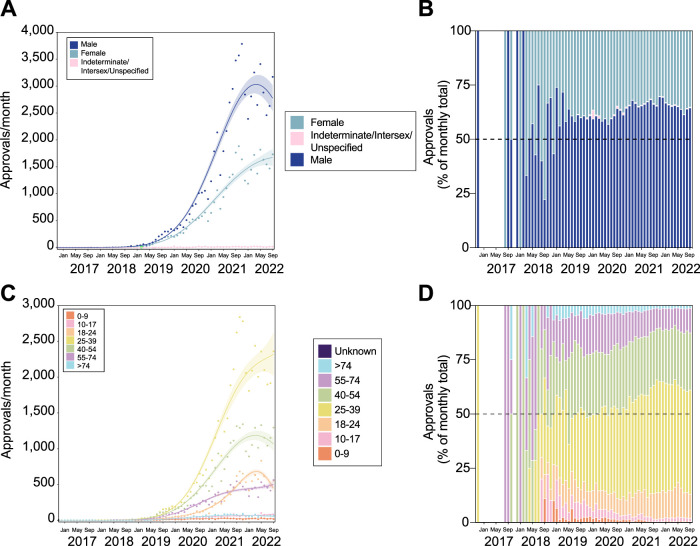
Patients receiving medicinal cannabis for psychiatric indications are predominantly younger and male. Trends in patient sex **(A,B)** and age **(C,D)**. Approval trends over time showing a recent decrease in the rate of approvals for males **(A)**, but continued growth in young patients, particularly aged 25–39 **(C)**. The proportion of these changes is also shown **(B,D)**, and suggests that while the number of male prescriptions may be decreasing, the relative proportion of prescribing remains relatively consistent. The lines of best fit in panels **(A,B)** are shown by the solid line with shaded area showing standard error of the mean. The gap in panels **(B,D)** indicates no applications submitted during this period.

Monthly numbers of SAS-B approvals for psychiatric indications in males grew significantly, but seems to be recently decreasing (2nd degree polynomial, R^2^ = 0.986, ∆m = 12,106.310; Figure 5A), while the rate of growth for females was less compared to males (3rd degree polynomial, R^2^ = 0.989, ∆m = 3.970). The proportion of prescribing for males has changed little over time following the initial prescription increase in November 2019 (Figure 5B).

The prescribing rate for patients aged 25–39 has grown sharply from November 2019, far outpacing any other group (4^th^ degree polynomial, R^2^ = 0.988, ∆m = 12.218; Figure 5C). Unsurprisingly, the proportion of approvals made up by this age group has increased over time, while the proportion of patients aged 55–74 and >74 has decreased (Figure 5D).

**FIGURE 6:**
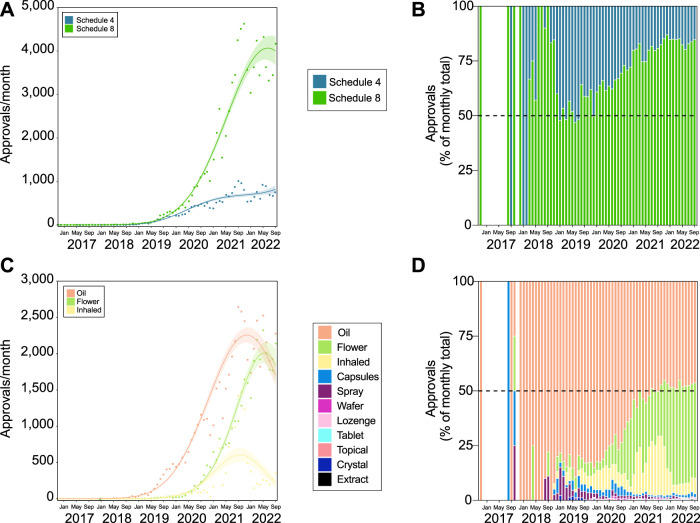
Approvals for medicinal cannabis products for the treatment of psychiatric indications are largely THC-containing oil or flower products. Trends in product schedule **(A,B)** and type **(C,D)**. Approval trends over time showing continued growth of S8 access **(A)**, which is reflected in the proportional access **(B)**. The rate of approvals for the top three product types (oil, flower, and inhaled) all appear to be decreasing **(C)**. However, oil and flower at least seem to have consistent proportional approvals **(D)**. The lines of best fit in panels **(A,B)** are shown by the solid line with shaded area showing standard error of the mean. The gap in panels **(B,D)** indicates no applications submitted during this period.

Prescribing of Schedule 4 products for psychiatric indications has grown slowly over time (3rd degree polynomial, R^2^ = 0.979, ∆m = 29.083; Figure 6A) compared with S8 (2nd degree polynomial, R^2^ = 0.987, ∆m = 14,260.970). Likewise, the proportion of S8 products approved for psychiatric indications has grown over time, though appears fairly uniform within the last few months (Figure 6B).

Approvals for oil products have rapidly increased over time, though has reduced in rate since the peak around September 2021 (2nd degree polynomial, R^2^ = 0.984, ∆m = 9,691.022; Figure 6C). Approvals for flower products followed a similar, but delayed trend, with peak around May 2022 (3rd degree polynomial, R^2^ = 0.991, ∆m = 10,422). Overall, the proportion of approvals for flower products has increased in recent years, and is now approaching that of oil products (Figure 6D).

## Discussion

The prescribing of unregistered medicinal cannabis products is a relatively new development in Australia that appears to have strong community support (Australian Institute of Health and Welfare, 2020) and attracts significant patient curiosity (Karanges et al., 2018). The current study shows that prescribing medicinal cannabis for psychiatric indications has gained significant momentum after a slow start. Although the general profile of patients with approvals for psychiatric indications is similar in some ways to the overall SAS-B dataset (predominantly male patients obtaining S8 products; MacPhail et al., 2022), this analysis reveals several important distinctions and recent trends that were not previously captured. The majority of patients who have SAS-B approval for psychiatric indications are younger, and are more likely to obtain flower products. The proportion of SAS-B approvals for males is greater for psychiatric conditions than for non-psychiatric indications. This pattern does not seem to match with proportional estimates of mental health conditions in Australia (Australian Institute of Health and Welfare, 2022), though does align with overall cannabis usage patterns (Australian Institute of Health and Welfare, 2020). Approvals for several psychiatric indication groups, including developmental disorders and depressive disorders, have increased substantially in recent times. The minimal number of approvals for the major psychiatric indications of schizophrenia, bipolar disorder, and obsessive-compulsive disorders (Australian Institute of Health and Welfare, 2022) was also particularly notable.

As outlined in recent systematic reviews and meta-analyses, the quality of evidence supporting the use of medicinal cannabis in psychiatric indications is patchy (Black et al., 2019; Bonaccorso et al., 2019; Hoch et al., 2019; Bahji et al., 2020; Khan et al., 2020; Sarris et al., 2020; Kloiber et al., 2021; Stanciu et al., 2021; Berger, Amminger, et al., 2022). Large RCTs with a low risk of bias are few and far between, and most clinical evidence has been gained from observational or retrospective cohort studies, open-label pilot trials, or laboratory studies. Such evidence often falls short of the standards that would be required for the formal registration of a new pharmaceutical entity by regulatory agencies (Black et al., 2019).

However, cannabis is not a novel pharmaceutical entity, having been used for millennia for therapeutic purposes. To add complexity, “medicinal cannabis” covers a diverse variety of cannabinoids with varying routes of administration, doses, and formulations. Recent systematic reviews, therefore, attempt to synthesize data from trials involving multiple conditions treated by diverse pharmaceutical and artisanal products. Consider, for example, the difference between a patient vaporizing a high dose of THC-containing cannabis flower to treat PTSD (a feasible option under the current SAS-B scheme) and another patient orally ingesting a moderate dose of a CBD-containing oil to treat generalized anxiety (also feasible). Both are “medicinal cannabis products” used for “anxiety” under the SAS-B, but their use, route of administration, and psychoactive effects are dramatically different. In this context, the available systematic reviews are an imperfect guide to optimal prescribing of the currently available products. Therefore, in contemplating whether the current prescribing of medicinal cannabis products in Australia for psychiatric conditions is rational and evidence-based, we must carefully dissect and interpret the evidence base, noting the limitations.

### Anxiety disorders

By far the largest number of psychiatry-related approvals in the present study are for anxiety disorders. In some ways, this represents an interesting ongoing experiment, given the limited current evidence for the anxiolytic effects of cannabinoids. Two recent systematic reviews and meta-analyses (Black et al., 2019; Stanciu et al., 2021) concluded there may be some evidence supporting efficacy for cannabinoids in treating anxiety, but that evidence was of very low quality, with a third analysis showing no effect when studies were corrected for publication bias (Bahji et al., 2020).

However, research in this space is rapidly evolving. An observational study of patients receiving CBD prescriptions in New Zealand for various conditions found a significant overall reduction of anxiety and improved quality of life in those prescribed CBD (dose range = 40–300 mg/day) for mental health conditions and non-cancer pain (Gulbransen et al., 2020). Two recent open-label trials have also reported significant effects: the first found a significant reduction in anxiety and comorbid depressive symptoms with 200–800 mg/day CBD in patients aged 12–25 with refractory anxiety (Berger, Li, et al., 2022), and the second showed positive effects in adults (ages 22–64) with moderate to severe anxiety with a CBD sublingual solution (dose range = 23–46 mg/day) (Dahlgren et al., 2022). These complement previous experimental clinical studies showing anxiolytic effects of CBD in healthy volunteers (Linares et al., 2019) and social phobia patients (Bergamaschi et al., 2011; Crippa et al., 2011) and in patients at high risk of developing psychosis (Appiah-Kusi et al., 2020). It is hoped that future and current clinical trials can clarify optimal dosing, products, and anxiety subtypes that might best benefit. The role of expectancy effects with CBD administration should also be clarified: a small study of adults undergoing an acute stress test showed the importance of *a priori* beliefs about the anxiolytic properties of CBD in determining outcomes (Spinella et al., 2021).

On the other hand, the widespread use of THC-containing S8 products in treating anxiety (Table 2) gives some grounds for concern, given that THC can reliably induce anxiety and paranoia in higher doses (Martin-Santos et al., 2012; Freeman et al., 2015). However, THC-induced anxiety may be obviated by the gradual up-titration of doses in patients, and by using oral low-dose formulations (also containing CBD) rather than smoking or vaporizing herbal cannabis. Outcomes such as this may be probed further in large registry studies currently underway in the UK (Project Twenty21) and Australia with patients being prescribed medicinal cannabis, including those using it for treatment of anxiety and PTSD (Drug Science, 2022; Sakal et al., 2022; Vickery et al., 2022).

### Trauma- and stressor-related disorders

Current evidence around the efficacy of cannabis and cannabinoid pharmaceuticals in PTSD has been reviewed recently in a focused fashion (Orsolini et al., 2019; Hindocha et al., 2020; Forsythe & Boileau, 2021; Rehman et al., 2021; Steardo et al., 2021; Sakal et al., 2022) and also included in the larger systematic reviews of psychiatric conditions published in the past 3 years (Black et al., 2019; Bonaccorso et al., 2019; Hoch et al., 2019; Botsford et al., 2020; Khan et al., 2020; Sarris et al., 2020; Kloiber et al., 2021; Stanciu et al., 2021). There are conspicuously high levels of self-medication with cannabis in patients with PTSD (Loflin et al., 2017), which is reported to provide symptomatic relief, particularly with respect to sleep and nightmares/flashbacks (Fraser, 2009; Passie et al., 2012); however, some studies suggest detrimental effects of cannabis use on PTSD symptom severity (Wilkinson et al., 2015). Studies of the pharmaceutical THC analogue nabilone have shown particular efficacy (Fraser, 2009; Cameron et al., 2014; Jetly et al., 2015), and some studies of THC/THC-predominant medicinal cannabis products have noted improved global functioning in PTSD (Mashiah, 2012; Roitman et al., 2014). The studies evaluating CBD only for PTSD symptom control are currently restricted to positive case reports (Shannon & Opila-Lehman, 2016; Elms et al., 2019), and a study on traumatic memory recall using a single administration of CBD (300 mg) showed little effect (Bolsoni et al., 2022). Again, we await larger well-controlled studies to validate current prescribing practice around CBD only products as well as those containing THC in PTSD, which appear to be ongoing (Telch et al., 2022).

### Insomnia and other sleep disorders

The existing evidence base supporting cannabinoids for the treatment of insomnia is limited. Recent systematic reviews (Suraev et al., 2020; Lavender et al., 2022) highlighted the limited evidence supporting cannabinoids in treating clinician-diagnosed insomnia disorder (as opposed to patient self-reported insomnia, or “sleep problems”). Most published RCTs of “insomnia” are often secondary to other conditions, such as chronic pain, and are only conducted over acute timelines (e.g., single dose to a maximum of 4 weeks), making conclusions about the longevity of the self-reported effects of cannabinoids uncertain. Current SAS-B prescribing for insomnia is predominantly for THC-containing products (Table 2), which arguably aligns with available evidence, although there are exceptions. Two RCTs of nabilone in patients with sleep issues secondary to chronic pain showed modest efficacy in improving total sleep time and efficiency (Ware et al., 2010; Zalai, 2015). However, one such study concluded that nabilone was not an effective option as it concurrently increased sleep onset latency (Zalai, 2015). A third RCT of THC observed a reduction in sleep latency but only evaluated the effects of a single acute dose (Cousens & DiMascio, 1973), while early results from another trial with a single 200 mg CBD and 10 mg THC administration suggested a decrease in total sleep time (Suraev et al., 2022). Similarly, a study in healthy volunteers reported no effect of THC alone on sleep parameters, and when combined with CBD (in the form of nabiximols) actually increased wakefulness (Nicholson et al., 2004). Finally, a recent RCT in adults with chronic insomnia reported improvements over 2 weeks in subjective sleep quality, sleep-onset latency, total sleep time, feeling of rest upon waking with nightly administration of used ZTL-101 (containing 10 mg THC, 1 mg cannabinol, and 0.5 mg CBD) (Walsh et al., 2021). The majority of support for THC prescribing in sleep disorders (including insomnia) seems to be from the community and patient self-report (Lintzeris et al., 2022) as opposed to a robust clinical evidence base (with the exception of one recent RCT), and this is reflected in the recommendations of the American Academy of Sleep Medicine (Ramar et al., 2018).

At present, there is no compelling rationale for prescribing CBD for chronic refractory insomnia. Evidence for the use of CBD in insomnia is limited to a retrospective case series (in patients with “poor sleep”) (Shannon et al., 2019) and a single acute dose self-report RCT (Carlini & Cunha, 1981), neither of which makes a strong case for long term CBD efficacy to support prescribing. Again, it is anticipated that evidence from these larger, longer duration studies will shed better light on the therapeutic use of cannabinoids for insomnia and sleep disorders.

### Neurodevelopmental disorders (ASD, ADHD)

Prescribing THC-containing products to children is controversial in any setting. Prescribing for psychiatric indications is particularly so, given the deleterious impacts of chronic THC exposure on the developing brain and adult behavioral phenotype that is routinely observed in animal models (Quinn et al., 2008; Trezza et al., 2008). Our analysis uncovers noteworthy prescribing of medicinal cannabis products to <18-year-olds largely divided between anxiety disorders and neurodevelopmental disorders (primarily ASD). While prescribing CBD in specific pediatric epilepsies is now evidence-based (Devinsky et al., 2017; Laux et al., 2019), the evidence for cannabinoid efficacy in conditions such as anxiety, ASD and ADHD is minimal. This is particularly true for ADHD–a single RCT in adults that concluded no significant effect of nabiximols treatment on cognitive performance and only suggestive effects on secondary hyperactivity measures (Cooper et al., 2017). There have been no studies evaluating CBD-only preparations in ADHD cohorts, despite this accounting for 15.9% of ADHD approvals under SAS-B (Table 2).

ASD attracts even more SAS-B prescribing than ADHD, yet the current evidence base consists of only a single RCT comparing a 20:1 CBD:THC whole extract, a purified isolate product at the same CBD:THC ratio, and placebo, in patients aged 5–21 years. Parent-reported measures of behavior did not reveal a significant effect over 12 weeks of either treatment compared with placebo, but clinical evaluation of disruptive behavior was improved with the extract product (Aran et al., 2021). Overall improvements in anxiety, sleep, and behavior remain inconsistent across case series and observational studies, with approximately one-third of children seemingly responding well (Efron, 2021; Fletcher et al., 2021). However, a significant caveat to these studies is their reliance on parental reports, which is notoriously variable and/or prone to bias. Notably, CBD-only prescribing accounts for 56.3% of ASD approvals, yet there are no published studies of CBD-only products in this patient cohort. Current CBD prescribing for ASD may be more reflective of caution around the use of THC products in children rather than being evidence-driven.

### Schizophrenia, bipolar disorder and depressive disorders

A major finding of the current study is the minimal SAS-B approvals for the major psychiatric disorders of schizophrenia and bipolar disorder. The prescribing for depression is rising dramatically of late, though it still represents a small proportion of the overall prescribing. Presumably, the uncertainty that surrounds a causal association between cannabis use and psychosis (Colizzi et al., 2020; D'Souza et al., 2022; Di Forti et al., 2019; Hill, 2015; National Academies of Sciences, 2017; Pasman et al., 2018) explains the high degree of caution in prescribing medicinal cannabis products for schizophrenia and bipolar disorder, although it does not necessarily account for the limited S4 (CBD-dominant) prescribing (*n* = 22). A number of moderate-quality RCTs, with well-sized patient cohorts, evaluating CBD-only products for the treatment of schizophrenia have shown positive outcomes (Leweke et al., 2012; Boggs et al., 2018; McGuire et al., 2018). The limited approvals for schizophrenia highlight a notable gap between the existing evidence base and prescribing decisions. Similarly, the only published study involving CBD for treating bipolar disorder produced equivocal findings and concluded that CBD was ineffective in treating mania (Zuardi et al., 2010), and yet 16.5% of approvals have been for CBD-only-containing products. Psychiatric prescribing practices in these disorders are interesting examples that highlight a potential disconnect between current prescribing and awareness of the current evidence base.

Finally, depression is a highly prevalent condition, with recent figures that more than 10% of Australians are prescribed antidepressant medications (Stephenson et al., 2013; Brett et al., 2017). Medicinal cannabis prescribing for depressive disorders is low relative to other psychiatric indications, but is on the rise. This may reflect the lack of any RCTs specifically focused on the treatment of depression with cannabinoids (Scherma et al., 2020; Tibbo et al., 2021), as well as documented positive associations between cannabis use and depression, albeit with uncertainty of causal direction (Horwood et al., 2012; Bahorik et al., 2017; Hodgson et al., 2020). The available evidence for medicinal cannabis in treating depression is of poor quality and is restricted to several positive case reports involving dronabinol (Blaas, 2008) and other anecdotal observations of unregulated cannabis use in patients with complex psychiatric histories (Gruber et al., 1996). To date, SAS-B approvals for depressive disorders are primarily S8 products (87.3%; Table 2). There is no current evidence to support CBD-only prescribing for depression.

One alternative possible explanation for the low number of approvals for depressive disorders could be the overlap with comorbid chronic pain, which is highly prevalent in depressive populations. However, it would not be captured in the SAS-B data (see MacPhail et al., 2022). Alternatively, the widespread use of medicinal cannabis products for anxiety disorders may also be inadvertently benefitting depression, which shows high co-morbidity with anxiety.

The growing use of medicinal cannabis can be seen as part of a broader movement within psychiatry toward use of unconventional therapies, or rather the return to some of the older options (including cannabis) ruled too radical in recent times, including a range of traditional “recreational” drugs, being accepted into clinical practice (Nutt, 2019). Examples include the use of ketamine and psilocybin for depression (Thomas et al., 2017; Rosenblat et al., 2019; Carhart-Harris et al., 2021; Goodwin et al., 2022) and MDMA for PTSD (Mitchell et al., 2021). This is perhaps a response to the obstinately dry pipeline of novel psychiatric medications from traditional pharmaceutical routes, and the ongoing use of traditional prescription psychotropics that are often older than the prescriber and the patient.

This is not to say that psychiatry should abandon caution and prescribe unregistered medicines as a first-line intervention. Indeed, official guidance on medicinal cannabis prescribing around psychiatric conditions is notably absent, meaning that clinicians have no readily available source of advice on rational prescribing for conditions such as anxiety, insomnia, PTSD and ASD. Prescribers struggle to find quality information to guide their use of the more than 360 medicinal cannabis products currently available under SAS-B within Australia. The TGA has produced guidance documents that outline the quality of the supportive clinical evidence for five different conditions: chronic non-cancer pain, epilepsy, multiple sclerosis, palliative care, and nausea and vomiting (Therapeutic Goods Administration, 2019). However, similar evidence-based guidance for psychiatric disorders is needed with some urgency, which is starting to be addressed by international peers at present yet has not been as much of a focus in the Australian context to date. Development of prescribing guidelines for MC has been completed by several groups in the UK, including NICE (National Institute for Health and Care Excellence, 2019) and the Medicinal Cannabis Clinicians Society (Medical Cannabis Clinicians’ Society, 2021). Yet, these are still very generalist in nature and do not provide guidance specific to psychiatric indications using evidence-based conclusions.

Nor does this lack of guidance suggest that supervised use of medicinal cannabis for these conditions should be completely halted. The reality is that even when legal access pathways are available, many Australian patients with mental health conditions report self-medicating with cannabis (Lintzeris et al., 2022), which is perhaps a reflection of the uneasiness of some healthcare practitioners in administering and supervising use for these indications (Karanges et al., 2018). Unsupervised use of medicinal cannabis (prescribed or otherwise) may come with risk, and non-disclosure can ultimately affect quality of care received by patients (Cairns & Kelly, 2017; Stuart-Maver, 2020). Indeed, medicinal cannabis use under strict supervision of a healthcare practitioner may currently be the best option to balance these risks while providing appropriate care, given the general tolerability and safety under supervised use (Vickery et al., 2022). Medicinal cannabis for treating psychiatric indications is a rapidly evolving field with new studies being published regularly, hopefully providing greater clarity in the near future.

## Conclusion

The purpose of this review was to present data on the current TGA approvals for medicinal cannabis products in Australia under the SAS-B scheme, their use for psychiatric indications, and to synthesize these data and relate back to the existing evidence base. We hope that our analysis will aid in transparency around current SAS-B prescribing practices in the psychiatric realm within Australia and stimulate further discussion, evaluation, and research into whether medicinal cannabis products represent effective standalone or adjunctive treatments for use within psychiatry. This issue is only likely to intensify in the coming months and years with the tremendous worldwide popularity and associated patient interest in using medicinal cannabis to treat a cornucopia of conditions. For this reason, the discussion must continue with the input of those in the academic and clinical community who are best placed to offer considered and balanced scientific views, with specific effort placed on facilitating high-quality RCTs, particularly where prescribing is disproportionate to existing clinical evidence of efficacy.

## Data Availability

Publicly available datasets were analyzed in this study. This data can be found here: https://www.tga.gov.au/sites/default/files/2022-12/foi-4020-01.XLSX.

## References

[B1] Abel E. L. (2005). Jacques joseph Moreau (1804-1884). Am. J. Psychiatry 162 (3), 458. 10.1176/appi.ajp.162.3.458 15741461

[B2] Appiah-Kusi E. Petros N. Wilson R. Colizzi M. Bossong M. G. Valmaggia L. (2020). Effects of short-term cannabidiol treatment on response to social stress in subjects at clinical high risk of developing psychosis. Psychopharmacol. Berl. 237, 1121–1130. 10.1007/s00213-019-05442-6 PMC7113209 31915861

[B3] Aran A. Harel M. Cassuto H. Polyansky L. Schnapp A. Wattad N. (2021). Cannabinoid treatment for autism: A proof-of-concept randomized trial. Mol. Autism 12 (1), 6. 10.1186/s13229-021-00420-2 33536055 PMC7860205

[B4] Arkell T. R. Lintzeris N. Kevin R. C. Ramaekers J. G. Vandrey R. Irwin C. (2019). Cannabidiol (CBD) content in vaporized cannabis does not prevent tetrahydrocannabinol (THC)-induced impairment of driving and cognition. Psychopharmacol. Berl. 236 (9), 2713–2724. 10.1007/s00213-019-05246-8 PMC6695367 31044290

[B5] Arseneault L. Cannon M. Poulton R. Murray R. Caspi A. Moffitt T. E. (2002). Cannabis use in adolescence and risk for adult psychosis: Longitudinal prospective study. BMJ 325 (7374), 1212–1213. 10.1136/bmj.325.7374.1212 12446537 PMC135493

[B6] Australian Bureau of Statistics (2022). Population: Census. Retrieved 22 December 2022 from https://www.abs.gov.au/statistics/people/population/population-census/2021

[B7] Australian Institute of Health and Welfare (2020). National drug strategy household survey 2019. https://www.aihw.gov.au/reports/illicit-use-of-drugs/national-drug-strategy-household-survey-2019.

[B8] Australian Institute of Health and Welfare (2022). Prevalence and impact of mental illness. https://www.aihw.gov.au/mental-health/overview/mental-illness.

[B9] Bahji A. Meyyappan A. C. Hawken E. R. (2020). Efficacy and acceptability of cannabinoids for anxiety disorders in adults: A systematic review & meta-analysis. J. Psychiatr. Res. 129, 257–264. 10.1016/j.jpsychires.2020.07.030 32827809

[B10] Bahorik A. L. Leibowitz A. Sterling S. A. Travis A. Weisner C. Satre D. D. (2017). Patterns of marijuana use among psychiatry patients with depression and its impact on recovery. J. Affect Disord. 213, 168–171. 10.1016/j.jad.2017.02.016 28242498 PMC5407687

[B11] Bartoń K. (2020). MuMIn: Multi-Model inference. https://CRAN.R-project.org/package=MuMIn.

[B12] Bawa Z. Saini B. McCartney D. Bedoya-Perez M. McLachlan A. J. McGregor I. S. (2022). A cross-sectional survey exploring the knowledge, experiences and attitudes of Australian pharmacists toward medicinal cannabis. Int. J. Clin. Pharm. 45, 375–386. 10.1007/s11096-022-01519-z 36446995 PMC9708126

[B13] Benson M. J. Abelev S. V. Corte C. J. Connor S. J. McGregor I. S. (2020). Attitudes and knowledge of Australian gastroenterologists around the use of medicinal cannabis for inflammatory bowel disease. Crohn's Colitis 360 (2), otaa045. 10.1093/crocol/otaa045 PMC9802365 36777304

[B14] Bergamaschi M. M. Queiroz R. H. Chagas M. H. de Oliveira D. C. De Martinis B. S. Kapczinski F. (2011). Cannabidiol reduces the anxiety induced by simulated public speaking in treatment-naive social phobia patients. Neuropsychopharmacology 36 (6), 1219–1226. 10.1038/npp.2011.6 21307846 PMC3079847

[B15] Berger M. Amminger G. P. McGregor I. S. (2022a). Medicinal cannabis for the treatment of anxiety disorders. Aust. J. Gen. Pract. 51 (8), 586–592. 10.31128/AJGP-04-21-5936 35908759

[B16] Berger M. Li E. Rice S. Davey C. G. Ratheesh A. Adams S. (2022b). Cannabidiol for treatment-resistant anxiety disorders in young people: An open-label trial. J. Clin. Psychiatry 83 (5), 21m14130. 10.4088/JCP.21m14130 35921510

[B17] Blaas K. (2008). Treating depression with cannabinoids. Cannabinoids 3 (2), 8–10. https://www.cannabis-med.org/data/pdf/en_2008_02_2_0.pdf.

[B18] Black N. Stockings E. Campbell G. Tran L. T. Zagic D. Hall W. D. (2019). Cannabinoids for the treatment of mental disorders and symptoms of mental disorders: A systematic review and meta-analysis. Lancet Psychiatry 6 (12), 995–1010. 10.1016/S2215-0366(19)30401-8 31672337 PMC6949116

[B19] Boggs D. L. Surti T. Gupta A. Gupta S. Niciu M. Pittman B. (2018). The effects of cannabidiol (CBD) on cognition and symptoms in outpatients with chronic schizophrenia a randomized placebo controlled trial. Psychopharmacol. Berl. 235 (7), 1923–1932. 10.1007/s00213-018-4885-9 29619533

[B20] Bolsoni L. M. Crippa J. A. S. Hallak J. E. C. Guimaraes F. S. Zuardi A. W. (2022). Effects of cannabidiol on symptoms induced by the recall of traumatic events in patients with posttraumatic stress disorder. Psychopharmacol. Berl. 239 (5), 1499–1507. 10.1007/s00213-021-06043-y 35029706

[B21] Bonaccorso S. Ricciardi A. Zangani C. Chiappini S. Schifano F. (2019). Cannabidiol (CBD) use in psychiatric disorders: A systematic review. Neurotoxicology 74, 282–298. 10.1016/j.neuro.2019.08.002 31412258

[B22] Botsford S. L. Yang S. George T. P. (2020). Cannabis and cannabinoids in mood and anxiety disorders: Impact on illness onset and course, and assessment of therapeutic potential. Am. J. Addict. 29 (1), 9–26. 10.1111/ajad.12963 31577377 PMC6925309

[B23] Brett J. Karanges E. A. Daniels B. Buckley N. A. Schneider C. Nassir A. (2017). Psychotropic medication use in Australia, 2007 to 2015: Changes in annual incidence, prevalence and treatment exposure. Aust. N. Z. J. Psychiatry 51 (10), 990–999. 10.1177/0004867417721018 28758432

[B24] Burnham K. P. Anderson D. R. (2002). A practical information-theoretic approach. Berlin, Germany: Springer.

[B25] Cairns E. A. Kelly M. E. M. (2017). Why support a separate medical access framework for cannabis? CMAJ 189 (28), E927-E928–E928. 10.1503/cmaj.170427 28716846 PMC5515644

[B26] Cameron C. Watson D. Robinson J. (2014). Use of a synthetic cannabinoid in a correctional population for posttraumatic stress disorder-related insomnia and nightmares, chronic pain, harm reduction, and other indications: A retrospective evaluation. J. Clin. Psychopharmacol. 34 (5), 559–564. 10.1097/JCP.0000000000000180 24987795 PMC4165471

[B27] Carhart-Harris R. Giribaldi B. Watts R. Baker-Jones M. Murphy-Beiner A. Murphy R. (2021). Trial of psilocybin versus escitalopram for depression. N. Engl. J. Med. 384 (15), 1402–1411. 10.1056/NEJMoa2032994 33852780

[B28] Carlini E. A. Cunha J. M. (1981). Hypnotic and antiepileptic effects of cannabidiol. J. Clin. Pharmacol. 21 (S1), 417S-427S–427S. 10.1002/j.1552-4604.1981.tb02622.x 7028792

[B29] Colizzi M. Ruggeri M. Bhattacharyya S. (2020). Unraveling the intoxicating and therapeutic effects of cannabis ingredients on psychosis and cognition. Front. Psychol. 11, 833. 10.3389/fpsyg.2020.00833 32528345 PMC7247841

[B30] Cooper R. E. Williams E. Seegobin S. Tye C. Kuntsi J. Asherson P. (2017). Cannabinoids in attention-deficit/hyperactivity disorder: A randomised-controlled trial. Eur. Neuropsychopharmacol. 27 (8), 795–808. 10.1016/j.euroneuro.2017.05.005 28576350

[B31] Cousens K. DiMascio A. (1973). (- Delta 9 THC as an hypnotic. An experimental study of three dose levels. Psychopharmacologia 33 (4), 355–364. 10.1007/BF00437513 4776660

[B32] Crippa J. A. Derenusson G. N. Ferrari T. B. Wichert-Ana L. Duran F. L. Martin-Santos R. (2011). Neural basis of anxiolytic effects of cannabidiol (CBD) in generalized social anxiety disorder: A preliminary report. J. Psychopharmacol. 25 (1), 121–130. 10.1177/0269881110379283 20829306

[B33] D'Souza D. C. DiForti M. Ganesh S. George T. P. Hall W. Hjorthoj C. (2022). Consensus paper of the WFSBP task force on cannabis, cannabinoids and psychosis. World J. Biol. Psychiatry 23 (10), 719–742. 10.1080/15622975.2022.2038797 35315315

[B34] Dahlgren M. K. Lambros A. M. Smith R. T. Sagar K. A. El-Abboud C. Gruber S. A. (2022). Clinical and cognitive improvement following full-spectrum, high-cannabidiol treatment for anxiety: Open-label data from a two-stage, phase 2 clinical trial. Commun. Med. (Lond) 2 (1), 139. 10.1038/s43856-022-00202-8 36352103 PMC9628346

[B35] DeLisi L. E. (2008). The effect of cannabis on the brain: Can it cause brain anomalies that lead to increased risk for schizophrenia? Curr. Opin. Psychiatry 21 (2), 140–150. 10.1097/YCO.0b013e3282f51266 18332661 PMC4337025

[B36] Devinsky O. Cross J. H. Laux L. Marsh E. Miller I. Nabbout R. (2017). Trial of cannabidiol for drug-resistant seizures in the Dravet syndrome. N. Engl. J. Med. 376 (21), 2011–2020. 10.1056/NEJMoa1611618 28538134

[B37] Devinsky O. Patel A. D. Cross J. H. Villanueva V. Wirrell E. C. Privitera M. (2018). Effect of cannabidiol on drop seizures in the lennox-gastaut syndrome. N. Engl. J. Med. 378 (20), 1888–1897. 10.1056/NEJMoa1714631 29768152

[B38] Di Forti M. Quattrone D. Freeman T. P. Tripoli G. Gayer-Anderson C. Quigley H. (2019). The contribution of cannabis use to variation in the incidence of psychotic disorder across europe (EU-GEI): A multicentre case-control study. Lancet Psychiatry 6 (5), 427–436. 10.1016/S2215-0366(19)30048-3 30902669 PMC7646282

[B39] Drug Science (2022). Access medical cannabis with project Twenty21. Retrieved 22 December 2022 from https://www.drugscience.org.uk/twenty21/

[B40] Efron D. (2021). Potential therapeutic uses of cannabinoids to treat behavioural problems in children and adolescents with developmental disorders. Aust. J. Gen. Pract. 50 (6), 352–355. 10.31128/AJGP-01-21-5809 34059838

[B41] Elms L. Shannon S. Hughes S. Lewis N. (2019). Cannabidiol in the treatment of post-traumatic stress disorder: A case series. J. Altern. Complement. Med. 25 (4), 392–397. 10.1089/acm.2018.0437 30543451 PMC6482919

[B42] Englund A. Oliver D. Chesney E. Chester L. Wilson J. Sovi S. (2022). Does cannabidiol make cannabis safer? A randomised, double-blind, cross-over trial of cannabis with four different CBD:THC ratios. Neuropsychopharmacology 48, 869–876. 10.1038/s41386-022-01478-z 36380220 PMC10156730

[B43] Fischer B. Robinson T. Bullen C. Curran V. Jutras-Aswad D. Medina-Mora M. E. (2022). Lower-risk cannabis use guidelines (lrcug) for reducing health harms from non-medical cannabis use: A comprehensive evidence and recommendations update. Int. J. Drug Policy 99, 103381. 10.1016/j.drugpo.2021.103381 34465496

[B44] Fletcher S. Pawliuk C. Ip A. Huh L. Rassekh S. R. Oberlander T. F. (2021). Medicinal cannabis in children and adolescents with autism spectrum disorder: A scoping review. Child. Care Health Dev. 48, 33–44. 10.1111/cch.12909 34403168

[B45] Forsythe M. L. Boileau A. J. (2021). Use of cannabinoids for the treatment of patients with post-traumatic stress disorder. J. Basic Clin. Physiol. Pharmacol. 33 (2), 121–132. 10.1515/jbcpp-2020-0279 33662194

[B46] Fraser G. A. (2009). The use of a synthetic cannabinoid in the management of treatment-resistant nightmares in posttraumatic stress disorder (PTSD). CNS Neurosci. Ther. 15 (1), 84–88. 10.1111/j.1755-5949.2008.00071.x 19228182 PMC6494011

[B47] Freeman A. M. Petrilli K. Lees R. Hindocha C. Mokrysz C. Curran H. V. (2019). How does cannabidiol (CBD) influence the acute effects of delta-9-tetrahydrocannabinol (THC) in humans? A systematic review. Neurosci. Biobehav Rev. 107, 696–712. 10.1016/j.neubiorev.2019.09.036 31580839

[B48] Freeman D. Dunn G. Murray R. M. Evans N. Lister R. Antley A. (2015). How cannabis causes paranoia: Using the intravenous administration of ∆9-tetrahydrocannabinol (THC) to identify key cognitive mechanisms leading to paranoia. Schizophr. Bull. 41 (2), 391–399. 10.1093/schbul/sbu098 25031222 PMC4332941

[B49] Freeman T. P. Hindocha C. Baio G. Shaban N. D. C. Thomas E. M. Astbury D. (2020). Cannabidiol for the treatment of cannabis use disorder: A phase 2a, double-blind, placebo-controlled, randomised, adaptive bayesian trial. Lancet Psychiatry 7 (10), 865–874. 10.1016/S2215-0366(20)30290-X 32735782 PMC7116091

[B50] Goodwin G. M. Aaronson S. T. Alvarez O. Arden P. C. Baker A. Bennett J. C. (2022). Single-dose psilocybin for a treatment-resistant episode of major depression. N. Engl. J. Med. 387 (18), 1637–1648. 10.1056/NEJMoa2206443 36322843

[B51] Gorfinkel L. R. Stohl M. Hasin D. (2020). Association of depression with past-month cannabis use among US adults aged 20 to 59 Years, 2005 to 2016. JAMA Netw. Open 3 (8), e2013802. 10.1001/jamanetworkopen.2020.13802 32809032 PMC7435337

[B52] Gruber A. J. Pope H. G. Brown M. E. (1996). Do patients use marijuana as an antidepressant? Depression 4 (2), 77–80. 10.1002/(SICI)1522-7162 9160645

[B53] Gulbransen G. Xu W. Arroll B. (2020). Cannabidiol prescription in clinical practice: An audit on the first 400 patients in New Zealand. BJGP Open 4. 10.3399/bjgpopen20X101010 PMC7330185 32019776

[B54] Hay G. L. Baracz S. J. Everett N. A. Roberts J. Costa P. A. Arnold J. C. (2018). Cannabidiol treatment reduces the motivation to self-administer methamphetamine and methamphetamine-primed relapse in rats. J. Psychopharmacol. 32 (12), 1369–1378. 10.1177/0269881118799954 30260267

[B55] Hill M. (2015). Perspective: Be clear about the real risks. Nature 525 (7570), S14. 10.1038/525S14a 26398733

[B56] Hindocha C. Cousijn J. Rall M. Bloomfield M. A. P. (2020). The effectiveness of cannabinoids in the treatment of posttraumatic stress disorder (PTSD): A systematic review. J. Dual Diagn 16 (1), 120–139. 10.1080/15504263.2019.1652380 31479625

[B57] Hoch E. Niemann D. von Keller R. Schneider M. Friemel C. M. Preuss U. W. (2019). How effective and safe is medical cannabis as a treatment of mental disorders? A systematic review. Eur. Arch. Psychiatry Clin. Neurosci. 269 (1), 87–105. 10.1007/s00406-019-00984-4 30706168 PMC6595000

[B58] Hodgson K. Coleman J. R. I. Hagenaars S. P. Purves K. L. Glanville K. Choi S. W. (2020). Major depressive disorder working group of the psychiatric genomics cannabis use, depression and self-harm: Phenotypic and genetic relationships. Addiction 115 (3), 482–492. 10.1111/add.14845 31833150

[B59] Horwood L. J. Fergusson D. M. Coffey C. Patton G. C. Tait R. Smart D. (2012). Cannabis and depression: An integrative data analysis of four australasian cohorts. Drug Alcohol Depend. 126 (3), 369–378. 10.1016/j.drugalcdep.2012.06.002 22749560

[B60] Hothorn T. Hornik K. van de Wiel M. A. Zeileis A. (2006). A lego system for conditional inference. Am. Statistician 60 (3), 257–263. 10.1198/000313006x118430

[B61] Hutten N. Arkell T. R. Vinckenbosch F. Schepers J. Kevin R. C. Theunissen E. L. (2022). Cannabis containing equivalent concentrations of delta-9-tetrahydrocannabinol (THC) and cannabidiol (CBD) induces less state anxiety than THC-dominant cannabis. Psychopharmacol. Berl. 239 (11), 3731–3741. 10.1007/s00213-022-06248-9 PMC9584997 36227352

[B62] Jetly R. Heber A. Fraser G. Boisvert D. (2015). The efficacy of nabilone, a synthetic cannabinoid, in the treatment of PTSD-associated nightmares: A preliminary randomized, double-blind, placebo-controlled cross-over design study. Psychoneuroendocrinology 51, 585–588. 10.1016/j.psyneuen.2014.11.002 25467221

[B63] Karanges E. A. Suraev A. Elias N. Manocha R. McGregor I. S. (2018). Knowledge and attitudes of Australian general practitioners towards medicinal cannabis: A cross-sectional survey. BMJ Open 8 (7), e022101. 10.1136/bmjopen-2018-022101 PMC6042562 29970456

[B64] Kassambara A. (2020). ggpubr: ‘ggplot2’ based publication ready plots. R package version 0.4.0. https://rpkgs.datanovia.com/ggpubr/.

[B65] Khan R. Naveed S. Mian N. Fida A. Raafey M. A. Aedma K. K. (2020). The therapeutic role of cannabidiol in mental health: A systematic review. J. Cannabis Res. 2 (1), 2. 10.1186/s42238-019-0012-y 33526132 PMC7819291

[B66] Kloiber S. Matheson J. Kim H. K. Le Foll B. (2021). “Cannabinoid drugs in mental health disorders,” in NeuroPsychopharmacotherapy, 1–35. 10.1007/978-3-319-56015-1_465-1

[B67] Laux L. C. Bebin E. M. Checketts D. Chez M. Flamini R. Marsh E. D. (2019). Long-term safety and efficacy of cannabidiol in children and adults with treatment resistant Lennox-Gastaut syndrome or Dravet syndrome: Expanded access program results. Epilepsy Res. 154, 13–20. 10.1016/j.eplepsyres.2019.03.015 31022635

[B68] Lavender I. McGregor I. S. Suraev A. Grunstein R. R. Hoyos C. M. (2022). Cannabinoids, insomnia, and other sleep disorders. Chest 162 (2), 452–465. 10.1016/j.chest.2022.04.151 35537535

[B69] Leweke F. M. Piomelli D. Pahlisch F. Muhl D. Gerth C. W. Hoyer C. (2012). Cannabidiol enhances anandamide signaling and alleviates psychotic symptoms of schizophrenia. Transl. Psychiatry 2, e94. 10.1038/tp.2012.15 22832859 PMC3316151

[B70] Linares I. M. Zuardi A. W. Pereira L. C. Queiroz R. H. Mechoulam R. Guimaraes F. S. (2019). Cannabidiol presents an inverted U-shaped dose-response curve in a simulated public speaking test. Braz. J. Psychiatry 41 (1), 9–14. 10.1590/1516-4446-2017-0015 30328956 PMC6781714

[B71] Lintzeris N. Mills L. Abelev S. V. Suraev A. Arnold J. C. McGregor I. S. (2022). Medical cannabis use in Australia: Consumer experiences from the online cannabis as medicine survey 2020 (CAMS-20). Harm Reduct. J. 19 (1), 88. 10.1186/s12954-022-00666-w 35907959 PMC9338505

[B72] Loflin M. Earleywine M. Bonn-Miller M. (2017). Medicinal versus recreational cannabis use: Patterns of cannabis use, alcohol use, and cued-arousal among veterans who screen positive for PTSD. Addict. Behav. 68, 18–23. 10.1016/j.addbeh.2017.01.008 28088054

[B73] MacPhail S. L. Bedoya-Perez M. A. Cohen R. Kotsirilos V. McGregor I. S. Cairns E. A. (2022). Medicinal cannabis prescribing in Australia: An analysis of trends over the first five years. Front. Pharmacol. 13, 885655. 10.3389/fphar.2022.885655 35620292 PMC9127064

[B74] Martin-Santos R. Crippa J. A. Batalla A. Bhattacharyya S. Atakan Z. Borgwardt S. (2012). Acute effects of a single, oral dose of d9-tetrahydrocannabinol (THC) and cannabidiol (CBD) administration in healthy volunteers. Curr. Pharm. Des. 18 (32), 4966–4979. 10.2174/138161212802884780 22716148

[B75] Masataka N. (2019). Anxiolytic effects of repeated cannabidiol treatment in teenagers with social anxiety disorders. Front. Psychol. 10, 2466. 10.3389/fpsyg.2019.02466 31787910 PMC6856203

[B76] Mashiah M. “Medical cannabis as a treatment for chronic combat PTSD,” in Proceedings of the Promising results in an open pilot study Patients Out of Time Conference, Tucson, Arizona, USA, April 2012. https://maps.org/research-archive/presentations/Mashiah-MotiApril27.pdf.

[B77] McGuire P. Robson P. Cubala W. J. Vasile D. Morrison P. D. Barron R. (2018). Cannabidiol (CBD) as an adjunctive therapy in schizophrenia: A multicenter randomized controlled trial. Am. J. Psychiatry 175 (3), 225–231. 10.1176/appi.ajp.2017.17030325 29241357

[B78] Medical Cannabis Clinicians’ Society (2021). Recommendations and guidance on medical cannabis under prescription. https://www.ukmccs.org/wp-content/uploads/2021/03/Recommendations-and-Guidance-on-Medical-Cannabis-under-Prescription-3rd-Edition-2021-reduc.pdf.

[B79] Mitchell J. M. Bogenschutz M. Lilienstein A. Harrison C. Kleiman S. Parker-Guilbert K. (2021). MDMA-Assisted therapy for severe PTSD: A randomized, double-blind, placebo-controlled phase 3 study. Nat. Med. 27 (6), 1025–1033. 10.1038/s41591-021-01336-3 33972795 PMC8205851

[B80] Moore D. S. Kirkland S. (2013). The basic practice of statistics. New York, NY, USA: W. H. Freeman.

[B81] National Academies of Sciences, E., and Medicine (2017). The health effects of cannabis and cannabinoids: The current state of evidence and recommendations for research. Washington, D.C., United States: National Academies Press. 28182367

[B82] National Institute for Health and Care Excellence (2019). Cannabis-based medicinal products. Retrieved 22 December 2022 from https://www.nice.org.uk/guidance/ng144 31909928

[B83] Nicholson A. N. Turner C. Stone B. M. Robson P. J. (2004). Effect of Delta-9-tetrahydrocannabinol and cannabidiol on nocturnal sleep and early-morning behavior in young adults. J. Clin. Psychopharmacol. 24 (3), 305–313. 10.1097/01.jcp.0000125688.05091.8f 15118485

[B84] Nutt D. (2019). Psychedelic drugs-a new era in psychiatry? Dialogues Clin. Neurosci. 21 (2), 139–147. 10.31887/DCNS.2019.21.2/dnutt 31636488 PMC6787540

[B85] Onaemo V. N. Fawehinmi T. O. D'Arcy C. (2021). Comorbid cannabis use disorder with major depression and generalized anxiety disorder: A systematic review with meta-analysis of nationally representative epidemiological surveys. J. Affect Disord. 281, 467–475. 10.1016/j.jad.2020.12.043 33360749

[B86] Orsolini L. Chiappini S. Volpe U. Berardis D. Latini R. Papanti G. D. (2019). Use of medicinal cannabis and synthetic cannabinoids in post-traumatic stress disorder (PTSD): A systematic review. Med. Kaunas. 55 (9), 525. 10.3390/medicina55090525 PMC6780141 31450833

[B87] Pasman J. A. Verweij K. J. H. Gerring Z. Stringer S. Sanchez-Roige S. Treur J. L. (2018). GWAS of lifetime cannabis use reveals new risk loci, genetic overlap with psychiatric traits, and a causal influence of schizophrenia. Nat. Neurosci. 21 (9), 1161–1170. 10.1038/s41593-018-0206-1 30150663 PMC6386176

[B88] Passie T. Emrich H. M. Karst M. Brandt S. D. Halpern J. H. (2012). Mitigation of post-traumatic stress symptoms by cannabis resin: A review of the clinical and neurobiological evidence. Drug Test. Analysis 4 (7-8), 649–659. 10.1002/dta.1377 22736575

[B89] Pauli C. S. Conroy M. Vanden Heuvel B. D. Park S. H. (2020). Cannabidiol drugs clinical trial outcomes and adverse effects. Front. Pharmacol. 11, 63. 10.3389/fphar.2020.00063 32161538 PMC7053164

[B90] Quinn H. R. Matsumoto I. Callaghan P. D. Long L. E. Arnold J. C. Gunasekaran N. (2008). Adolescent rats find repeated Delta(9)-THC less aversive than adult rats but display greater residual cognitive deficits and changes in hippocampal protein expression following exposure. Neuropsychopharmacology 33 (5), 1113–1126. 10.1038/sj.npp.1301475 17581536

[B91] R Core Team (2022). “R: A language and environment for statistical computing,” in R foundation for statistical computing (Vienna, Austria: R Core Team). https://www.R-project.org/.

[B92] Ramar K. Rosen I. M. Kirsch D. B. Chervin R. D. Carden K. A. Aurora R. N. (2018). Medical cannabis and the treatment of obstructive sleep apnea: An American Academy of sleep medicine position statement. J. Clin. Sleep. Med. 14 (4), 679–681. 10.5664/jcsm.7070 29609727 PMC5886446

[B93] Rehman Y. Saini A. Huang S. Sood E. Gill R. Yanikomeroglu S. (2021). Cannabis in the management of PTSD: A systematic review. AIMS Neurosci. 8 (3), 414–434. 10.3934/Neuroscience.2021022 34183989 PMC8222769

[B94] Roitman P. Mechoulam R. Cooper-Kazaz R. Shalev A. (2014). Preliminary, open-label, pilot study of add-on oral Δ9-tetrahydrocannabinol in chronic post-traumatic stress disorder. Clin. Drug Investig. 34 (8), 587–591. 10.1007/s40261-014-0212-3 24935052

[B95] Rosenblat J. D. Carvalho A. F. Li M. Lee Y. Subramanieapillai M. McIntyre R. S. (2019). Oral ketamine for depression: A systematic review. J. Clin. Psychiatry 80 (3), 18r12475. 10.4088/JCP.18r12475 30995364

[B96] Sakal C. Lynskey M. Schlag A. K. Nutt D. J. (2022). Developing a real-world evidence base for prescribed cannabis in the United Kingdom: Preliminary findings from project Twenty21. Psychopharmacol. Berl. 239 (5), 1147–1155. 10.1007/s00213-021-05855-2 33970291

[B97] Sarris J. Sinclair J. Karamacoska D. Davidson M. Firth J. (2020). Medicinal cannabis for psychiatric disorders: A clinically-focused systematic review. BMC Psychiatry 20 (1), 24. 10.1186/s12888-019-2409-8 31948424 PMC6966847

[B98] Scherma M. Muntoni A. L. Riedel G. Fratta W. Fadda P. (2020). Cannabinoids and their therapeutic applications in mental disorders. Dialogues Clin. Neurosci. 22 (3), 271–279. 10.31887/DCNS.2020.22.3/pfadda 33162770 PMC7605020

[B99] Shannon S. Lewis N. Lee H. Hughes S. (2019). Cannabidiol in anxiety and sleep: A large case series. Perm. J. 23, 18–041. 10.7812/TPP/18-041 PMC6326553 30624194

[B100] Shannon S. Opila-Lehman J. (2016). Effectiveness of cannabidiol oil for pediatric anxiety and insomnia as part of posttraumatic stress disorder: A case report. Perm. J. 20 (4), 16–005. 10.7812/TPP/16-005 PMC5101100 27768570

[B101] Spinella T. C. Stewart S. H. Naugler J. Yakovenko I. Barrett S. P. (2021). Evaluating cannabidiol (CBD) expectancy effects on acute stress and anxiety in healthy adults: A randomized crossover study. Psychopharmacol. Berl. 238 (7), 1965–1977. 10.1007/s00213-021-05823-w PMC8233292 33813611

[B102] Stanciu C. N. Brunette M. F. Teja N. Budney A. J. (2021). Evidence for use of cannabinoids in mood disorders, anxiety disorders, and PTSD: A systematic review. Psychiatr. Serv. 72 (4), 429–436. 10.1176/appi.ps.202000189 33530732 PMC8857699

[B103] Steardo L. Jr. Carbone E. A. Menculini G. Moretti P. Steardo L. Tortorella A. (2021). Endocannabinoid system as therapeutic target of PTSD: A systematic review. Life (Basel) 11 (3), 214. 10.3390/life11030214 33803374 PMC8000573

[B104] Stephenson C. P. Karanges E. McGregor I. S. (2013). Trends in the utilisation of psychotropic medications in Australia from 2000 to 2011. Aust. N. Z. J. Psychiatry 47 (1), 74–87. 10.1177/0004867412466595 23144164

[B105] Stuart-Maver S. L. (2020). Working with clients who self-medicate using cannabis: Ethical and clinical considerations for psychologists. Prof. Psychol. Res. Pract. 51 (1), 77–84. 10.1037/pro0000269

[B106] Suraev A. McGregor I. Marshall N. D'Rozario A. Kao C. Gordon C. (2022). O006 acute effects of combined cannabidiol (CBD) and ∆9-tetrahydrocannabinol (THC) in insomnia disorder: A randomised, placebo-controlled trial using high-density eeg. Sleep. Adv. 3, A3–A4. 10.1093/sleepadvances/zpac029.005

[B107] Suraev A. S. Marshall N. S. Vandrey R. McCartney D. Benson M. J. McGregor I. S. (2020). Cannabinoid therapies in the management of sleep disorders: A systematic review of preclinical and clinical studies. Sleep. Med. Rev. 53, 101339. 10.1016/j.smrv.2020.101339 32603954

[B108] Telch M. J. Fischer C. M. Zaizar E. D. Rubin M. Papini S. (2022). Use of Cannabidiol (CBD) oil in the treatment of PTSD: Study design and rationale for a placebo-controlled randomized clinical trial. Contemp. Clin. Trials 122, 106933. 10.1016/j.cct.2022.106933 36154908

[B109] Therapeutic Goods Administration (2019). Medicinal cannabis - guidance documents. https://www.tga.gov.au/medicinal-cannabis-guidance-documents.

[B110] Therapeutic Goods Administration (2022a). Medicinal cannabis authorised prescriber scheme data. Retrieved 6 May 2023 from https://www.tga.gov.au/products/unapproved-therapeutic-goods/medicinal-cannabis-hub/medicinal-cannabis-access-pathways-and-patient-access-data/medicinal-cannabis-authorised-prescriber-scheme-data

[B111] Therapeutic Goods Administration (2022b). Medicinal cannabis products by active ingredients. Retrieved 22 December 2022 from https://www.tga.gov.au/medicinal-cannabis-products-active-ingredient

[B112] Thoen E. (2020). padr: Quickly get datetime data ready for analysis. https://github.com/EdwinTh/padr.

[B113] Thomas K. Malcolm B. Lastra D. (2017). Psilocybin-assisted therapy: A review of a novel treatment for psychiatric disorders. J. Psychoact. Drugs 49 (5), 446–455. 10.1080/02791072.2017.1320734 28481178

[B114] Tibbo P. G. McKee K. A. Meyer J. H. Crocker C. E. Aitchison K. J. Lam R. W. (2021). Are there therapeutic benefits of cannabinoid products in adult mental illness? Can. J. Psychiatry 66 (2), 185–194. 10.1177/0706743720945525 32911977 PMC7918871

[B115] Trezza V. Cuomo V. Vanderschuren L. J. (2008). Cannabis and the developing brain: Insights from behavior. Eur. J. Pharmacol. 585 (2-3), 441–452. 10.1016/j.ejphar.2008.01.058 18413273

[B116] Turna J. Syan S. K. Frey B. N. Rush B. Costello M. J. Weiss M. (2019). Cannabidiol as a novel candidate alcohol use disorder pharmacotherapy: A systematic review. Alcohol Clin. Exp. Res. 43 (4), 550–563. 10.1111/acer.13964 30698831 PMC6910215

[B117] Vaissie P. Monge A. Husson F. (2021). Factoshiny: Perform factorial analysis from 'FactoMineR' with a shiny application. https://CRAN.R-project.org/package=Factoshiny.

[B118] Venables W. N. Ripley B. D. (2002). Modern applied statistics with S. 4 ed. Berlin, Germany: Springer.

[B119] Vickery A. W. Roth S. Ernenwein T. Kennedy J. Washer P. (2022). A large Australian longitudinal cohort registry demonstrates sustained safety and efficacy of oral medicinal cannabis for at least two years. PLoS One 17 (11), e0272241. 10.1371/journal.pone.0272241 36399463 PMC9674134

[B120] Walsh J. H. Maddison K. J. Rankin T. Murray K. McArdle N. Ree M. J. (2021). Treating insomnia symptoms with medicinal cannabis: A randomized, crossover trial of the efficacy of a cannabinoid medicine compared with placebo. Sleep 44 (11), zsab149. 10.1093/sleep/zsab149 34115851 PMC8598183

[B121] Ware M. A. Fitzcharles M. A. Joseph L. Shir Y. (2010). The effects of nabilone on sleep in fibromyalgia: Results of a randomized controlled trial. Anesth. Analg. 110 (2), 604–610. 10.1213/ANE.0b013e3181c76f70 20007734

[B122] Weiland B. J. Thayer R. E. Depue B. E. Sabbineni A. Bryan A. D. Hutchison K. E. (2015). Daily marijuana use is not associated with brain morphometric measures in adolescents or adults. J. Neurosci. 35 (4), 1505–1512. 10.1523/JNEUROSCI.2946-14.2015 25632127 PMC4308597

[B123] Wickham H. Averick M. Bryan J. Chang W. McGowan L. François R. (2019). Welcome to the tidyverse. J. Open Source Softw. 4 (43), 1686. 10.21105/joss.01686

[B124] Wickham H. François R. Henry L. Müller K. (2021). dplyr: a grammar of data manipulation. https://dplyr.tidyverse.org/reference/dplyr-package.html.

[B125] Wickham H. (2016). ggplot2: Elegant graphics for data analysis. Berlin, Germany: Springer.

[B126] Wilke C. (2020). cowplot: streamlined plot theme and plot annotations for ‘ggplot2’. https://wilkelab.org/cowplot/.

[B127] Wilkinson S. T. Stefanovics E. Rosenheck R. A. (2015). Marijuana use is associated with worse outcomes in symptom severity and violent behavior in patients with posttraumatic stress disorder. J. Clin. Psychiatry 76 (9), 1174–1180. 10.4088/JCP.14m09475 26455669 PMC6258013

[B128] Zalai D. C. Hussain N. Shapiro C. M. (2015). Does cannabinoid really improve sleep? Testing the sleep effects of nabilone in chronic pain patients: A placebo-controlled, randomized, pilot study. Psychother. Psychosom. 84, 81. 10.1159/000438780

[B129] Zamarripa C. A. Spindle T. R. Surujunarain R. Weerts E. M. Bansal S. Unadkat J. D. (2023). Assessment of orally administered d9-tetrahydrocannabinol when coadministered with cannabidiol on d9-tetrahydrocannabinol pharmacokinetics and pharmacodynamics in healthy adults: A randomized clinical trial. JAMA Netw. Open 6 (2), e2254752. 10.1001/jamanetworkopen.2022.54752 36780161 PMC9926328

[B130] Zuardi A. Crippa J. Dursun S. Morais S. Vilela J. Sanches R. (2010). Cannabidiol was ineffective for manic episode of bipolar affective disorder. J. Psychopharmacol. 24 (1), 135–137. 10.1177/0269881108096521 18801823

